# Fused ring effect on optical nonlinearity and structure property relationship of anthracenyl chalcone based push-pull chromophores

**DOI:** 10.1371/journal.pone.0257808

**Published:** 2021-09-28

**Authors:** Dian Alwani Zainuri, Mundzir Abdullah, Muhamad Fikri Zaini, Hazri Bakhtiar, Suhana Arshad, Ibrahim Abdul Razak

**Affiliations:** 1 X-ray Crystallography Unit, School of Physics, Universiti Sains Malaysia, Penang, Malaysia; 2 Institute of Nano Optoelectronics Research and Technology (INOR), Universiti Sains Malaysia, Penang, Malaysia; 3 Department of Physics, Faculty of Sciences, Universiti Teknologi Malaysia, Johor Bahru, Johor, Malaysia; Nazarbayev University, KAZAKHSTAN

## Abstract

The Ultraviolet-visible (UV-Vis) spectra indicate that anthracenyl chalcones (ACs) have high maximum wavelengths and good transparency windows for optical applications and are suitable for optoelectronic applications owing to their HOMO–LUMO energy gaps (2.93 and 2.76 eV). Different donor substituents on the AC affect their dipole moments and nonlinear optical (NLO) responses. The positive, negative, and neutral electrostatic potential regions of the molecules were identified using molecular electrostatic potential (MEP). The stability of the molecule on account of hyperconjugative interactions and accompanying charge delocalization was analyzed using natural bond orbital (NBO) analysis. Open and closed aperture Z-scans were performed using a continuous-wave frequency-doubled diode-pumped solid-state (DPSS) laser to measure the nonlinear absorption and nonlinear refractive index coefficients, respectively. The valley-to-peak profile of AC indicated a negative nonlinear refractive index coefficient. The obtained single crystals possess reverse saturation absorption due to excited-state absorption. The structural and nonlinear optical properties of the molecules have been discussed, along with the role of anthracene substitution for enhancing the nonlinear optical properties. The calculated third-order susceptibility value was 1.10 x10^-4^ esu at an intensity of 4.1 kW/cm^2^, higher than the reported values for related chalcone derivatives. The NLO response for both ACs offers excellent potential in optical switching and limiting applications.

## Introduction

Nonlinear optics is a key area in science and technology for modern hi-tech applications. Organic materials have delocalized electrons in the n→π* or π→π* orbitals resulting in better nonlinear optical (NLO) properties than inorganic materials [1–4]; further, they have attracted the continued interest of researchers to develop better organic nonlinear optical materials [5]. Chalcones are donor-acceptor-conjugated organic materials with electron donor (D) and electron acceptor (A) groups linked by π-conjugated bridges, and they exhibit outstanding NLO properties [6–8].

**Fig 1:**
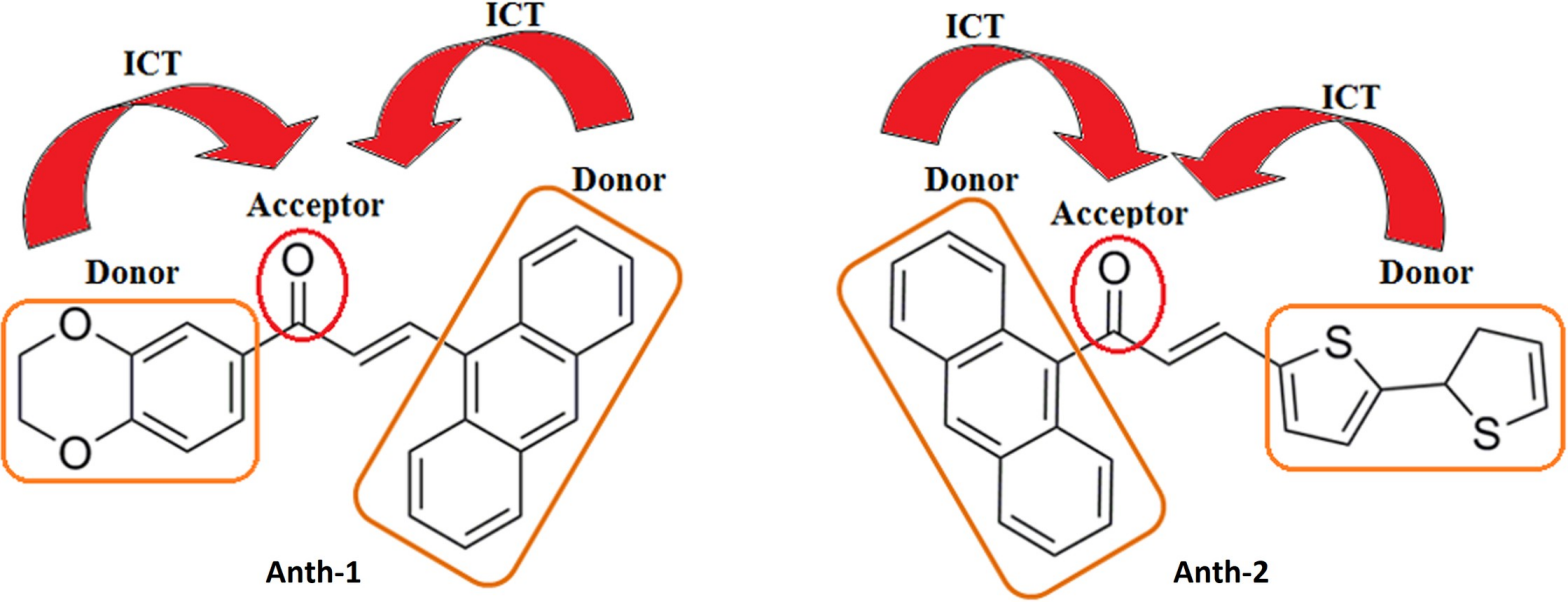
Intermolecular charge transfer between the AC.

Anthracene and its derivatives have several applications, including luminescent chemosensors and switches [9]. Recently, the optical nonlinearity of anthracene on account of its delocalized π-electron system has been reported [10, 11]. Additionally, anthracenyl-chalcones (AC) fulfill the three essential features for high nonlinear activity in an organic compound: a strong electron donor, a highly polarizable π-conjugated bridged, and a strong electron acceptor [12]. The intermolecular charge transfer (ICT) from the donor to the acceptor group via the anthracene chromophore plays a crucial role in the nonlinear absorption of the AC (Fig 1). The strong donor-acceptor conjugated interaction improves the push-pull functionalization and enhances the third-order optical nonlinearity of AC [13].

Several D–π–D and D–π–A molecules have been designed over the last three decades [10]. New systems with high charge transfer are required for high-efficiency third-order NLO because intramolecular charge transfer between the donor and acceptor yields good optical properties [14]. The linear D–π–A–π–D molecular architecture has attracted significant attention because of the reduced energy band gap of the resultant molecule via intermolecular charge transfer (ICT) between the donor and acceptor moieties [15]. Additionally, few structural characteristics, such as D–A strength, D–A relative position, and conjugation length, can also change the NLO properties [16, 17].

In order to evaluate the role of a highly conjugated electron-donating substituent (anthracene) on either side of the molecule, we synthesized two AC derivatives by modifying a previous study [18]. We obtained (*E*)-3-(anthracen-9-yl)-1-(2,3-dihydrobenzo[b][1,4]dioxin-6-yl)prop-2-en-1-one (**Anth-1**) and (*E*)-1-(anthracen-9-yl)-3-(2’,3’-dihydro-[2,2’-bithiophen]-5-yl)prop-2-en-1-one (**Anth-2**) with a D–π–A–D system with attached substituents. We studied the relationship between their structure and property using the X-ray structural analysis, infrared vibrational spectra, and Ultraviolet-visible (UV-vis) characterization of the AC derivatives. Additionally, theoretical calculations were done using density functional theory (DFT) to understand the physical, chemical, and electronic properties. The coefficients of nonlinear absorption, nonlinear refraction, and nonlinear optical susceptibility under continuous excitation conditions were determined using the Z-scan technique.

## Materials and methods

### Synthesis of chalcones: Claisen-schmidt reaction

**Scheme 1:**
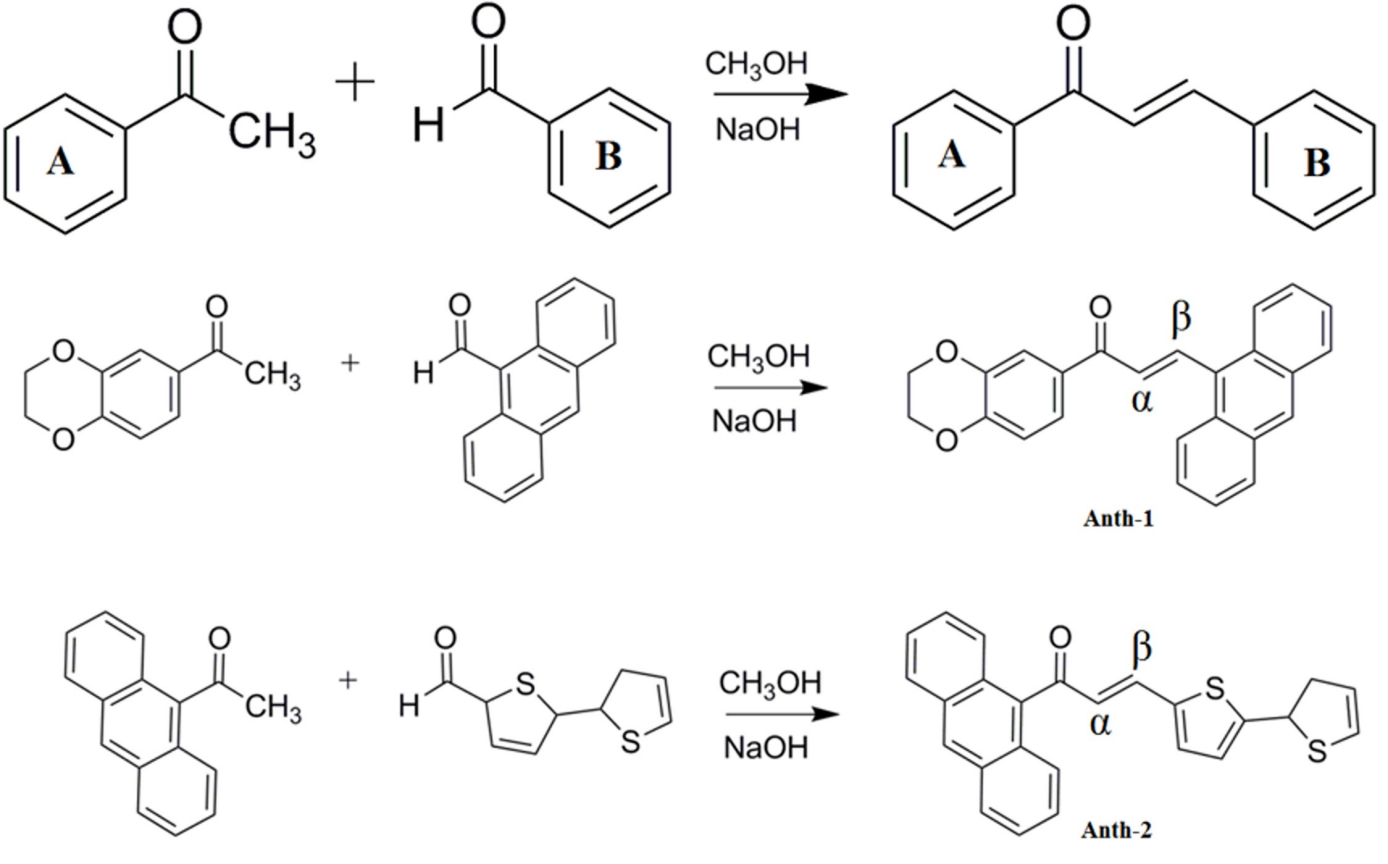
Synthesis of compounds.

**Fig 2:**
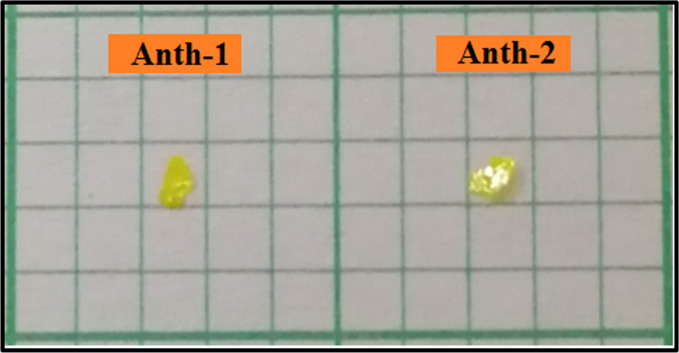
Single crystals of the Anth-1 and Anth-2.

Two chalcone derivatives with D-A substituents were prepared using the Claisen–Schmidt condensation reaction (Scheme 1). The compounds were prepared using a mixture of 1,4-Benzodioxan-6-yl methyl ketone (0.5 mmol, 0.08 g) and anthracene-9-carbaldehyde (0.5 mmol, 0.11 g) for **Anth-1,** and 9-acetylanthracene (0.5 mmol, 0.11 g) and 2,2’-Bithiophene-5-carbaldehyde (0.5 mmol, 0.09 g) for **Anth-2** in methanol with catalytic amount of NaOH (20%, 5 ml). The reaction mixture was stirred for 5–6 h at 25°C and subsequently poured into ice-cold water. The precipitate formed was collected by filtration, and the crude product was purified by repeated recrystallization. Single crystals were grown using the solvent evaporation technique with acetone as the solvent. The yellow, plate-shaped crystals of both compounds are shown in Fig 2.

### X-ray single crystal analysis

Single crystal X-ray diffraction (XRD) was performed using an Apex II Duo CCD area-detector diffractometer with MoKα radiation (λ = 0.71073 Å). Data collection was performed using APEX2 [19], whereas cell refinement and data reduction were performed using SAINT [19]. The crystal structure was solved directly using SHELXTL [20] and refined using the full-matrix least-squares technique on *F*^2^. Absorption correction of the final crystal data was performed using SADABS [19]. All geometrical calculations were performed using PLATON [21], and the molecular graphics were drawn using SHELXTL [20]. Anisotropic thermal factors were assigned to all the non-hydrogen atoms. The hydrogen atoms bound to carbon were positioned geometrically (C–H = 0.93 Å) with U_iso_(H) = 1.2 U_eq_ (C). Details of the data collection conditions and parameters of the refinement process are given in Table 1.

**Table 1:** Crystal data and structure refinement.

Compound	Anth-1	Anth-2
**CCDC**	1954101	1954102
**Molecular formula**	C_25_H_18_O_3_	C_25_H_16_OS_2_
**Molecular weight**	366.39	396.50
**Crystal system**	Monoclinic	Orthorhombic
**Space group**	*P*2_1_	*Pbca*
***a*/Å**	5.4388 (7)	14.5564 (11)
***b*/Å**	17.080 (2)	16.1318 (12)
***c*/Å**	10.0539 (12)	16.5002 (12)
***V*/ Å** ^**3**^	907.28 (19)	3874.6(5)
**Z**	2	8
***D***_**calc**_ **(Mg m** ^**−3**^ **)**	1.341	1.359
**Crystal Dimensions (mm)**	0.32 x 0.18 x 0.11	0.62 x 0.48 x 0.25
**μ/mm** ^**−1**^	0.09	0.29
**Radiation λ (Å)**	Mo kα	Mo kα
***F*(000)**	384	1648
**Reflections measured**	16502	193894
**Ranges/indices (*h*, *k*, *l*)**	-7 ≤ *h* ≤ 7	-21 ≤ *h* ≤ 21
	-24 ≤ *k* ≤ 24	-24 ≤ *k* ≤ 24
	-14 ≤ *l* ≤ 14	-24 ≤ *l* ≤ 24
**θ limit (°)**	2.1–30.3	2.3–32.2
**Unique reflections**	5377	6803
**Observed reflections (*I*> 2σ(*I*))**	3504	4814
**Parameters**	253	253
***R1 [a]*, *wR2 [I ≥ 2σ(I)] [b]*, *S***	0.048, 0.135, 1.02	0.050, 0.179, 1.00
***R***_**int**_	0.031	0.044

*w* = 1/[*σ*^2^ (*F*_o_^2^) + (0.0699*P*) ^2^] (Anth-1); 1/[*σ*^2^ (*F*_o_^2^) + (0.0961) ^2^+ 1.4694*P*] (Anth-2), where *P* = (*F*_o_^2^ + 2*F*_c_^2^)/3; ^[a]^ *R* = Σ||*F*_*o*_|–|*F*_*c*_||/Σ|*F*_*o*_|, ^[b]^ *R*_w_ = {*w*Σ(|*F*_*o*_|–|*F*_*c*_|)^2^/Σ*w*|*F*_*o*_|^2^}}^1/2^.

### Spectroscopy analyses

Fourier transform infrared spectroscopy (FTIR) spectra were recorded using a Fourier transform infrared attenuated total reflectance spectroscope (FTIR-ATR) (Perkin Elmer Spotlight 2000) equipped with a diamond crystal accessory. ^1^H and ^13^C nuclear magnetic resonance (NMR) spectra were recorded at 500 MHz in DMSO-d6 using a Bruker 500 MHz Avance III spectrometer. The chemical shifts (δ) are reported in parts per million (ppm) downfield from tetramethylsilane (TMS) internal reference. The UV-Vis absorption spectrum of the sample was recorded in Dimethylsulfoxide (DMSO) solution using a Shimadzu UV-1800 spectrophotometer in the spectral region of 200–800 nm.

### Third order nonlinearity

**Fig 3:**
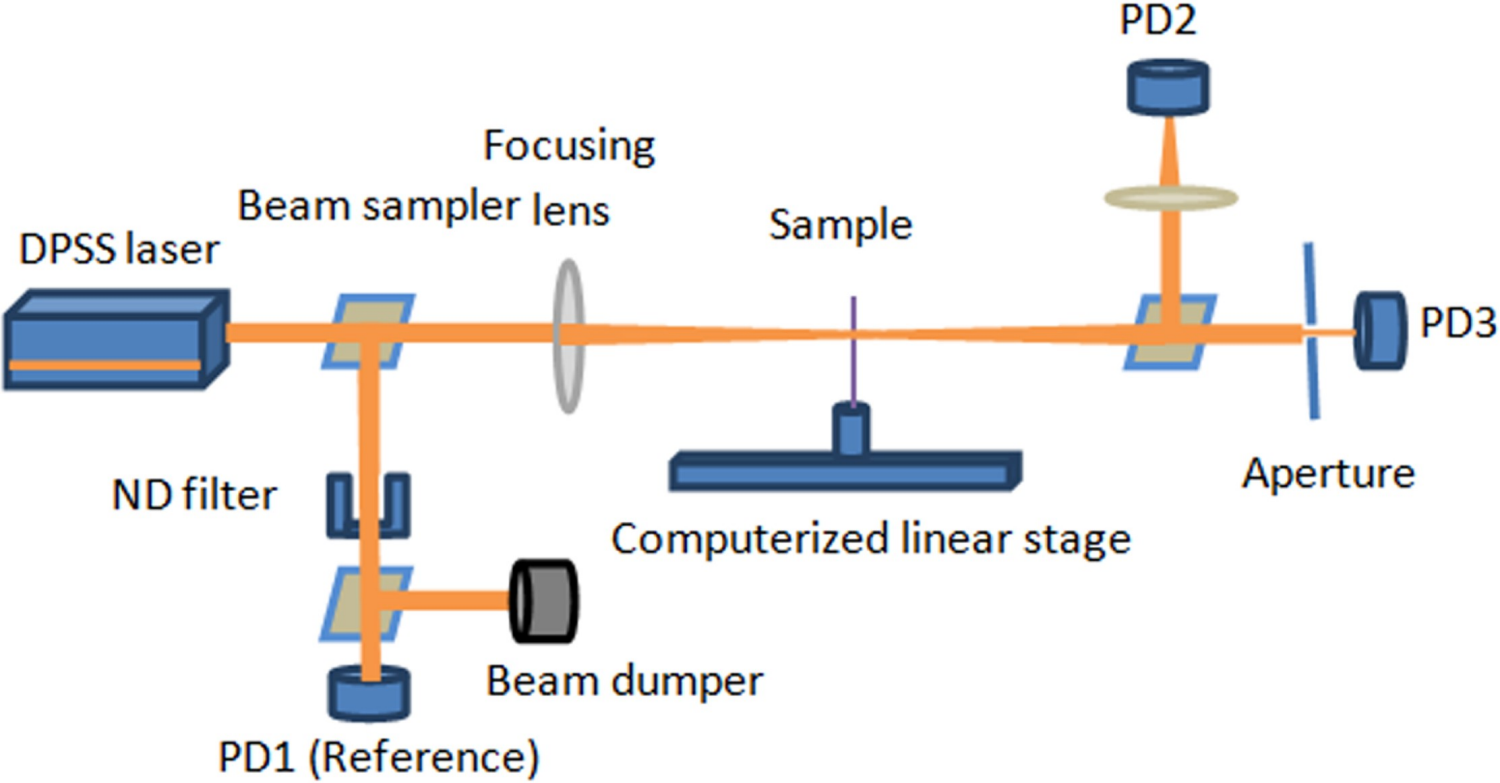
Z-scan technique setup.

The refractive index, *n*, of the samples was measured using a digital refractometer (DR201-95, KRUSS). UV-Vis absorbance spectra of both samples were obtained to calculate the linear absorption coefficient, *α*, with DMSO solvent. The NLO properties of the structure were investigated using the Z-scan technique, which computes the intensity-dependent transmission used to detect nonlinear refractions and nonlinear absorption simultaneously. The Z-scan setup is illustrated in Fig 3.

A continuous-wave frequency-doubled diode-pumped solid-state (DPSS) laser was used as the excitation laser, and a silicon-amplified photodetector with an adjustable gain was used as the detector. The radius of the Gaussian beam spot at the focal point was found to be 23 μm using a laser beam profiler (Beam Master, Coherent) with a converging lens of f = 20 cm. The sample was kept in a quartz cuvette of 1 mm path length and mounted on a precision motorized stage. Although the NLO coefficient of organic solvents, such as DMSO, is low (10^−14^–10^−16^ esu) [22], Z-scan measurements were performed using DMSO at several laser intensities to remove the NLO contribution of the solvent in the results. These scans did not reveal nonlinear signals, indicating that the nonlinear optical properties of the samples originate from the AC compounds.

The peak followed by a valley-normalized transmittance, obtained from the closed-aperture Z-scan, is defined as Δ*T*_*P-V*_ = *T*_*P*_−*T*_*v*_ [23]. The variation in this quantity as a function of |Δ*φ*_0_| is given by

$$\Delta T_{p-v}=0.406{\left(1-s\right)}^{0.25}|\Delta \varphi_{0}|$$ (1)

where,

|Δ*φ*_0_| = on-axis phase shift at the focus

*S* = the aperture linear transmittance [23] with the formula

S=1−exp(−2r02ω02) (2)

where,

*r*_0_ = Aperture radius

*ω*_0_ = beam radius at the aperture in the linear regime.

Then nonlinear refractive index, *n*_2_ [23] is given by

$$n_{2}=\frac{\Delta \varphi_{0}\lambda }{2\pi l_{0}L_{eff}}$$ (3)

where,

λ = wavelength of the laser

*I*_0_ = intensity of the laser beam at focus *z* = 0 [23]

$$L_{eff}=\frac{\left[1-exp(-\alpha L)\right]}{\alpha }$$ (4)

where,

*L*_*eff*_ = effective thickness of sample

*α* = linear absorption coefficient.

*L* = Thickness of the sample.

The nonlinear absorption coefficient, *β*, can be estimated using the open-aperture Z-scan data. The normalized transmittance for the open-aperture condition [24] is given by

$$T(z,s=1)=\sum_{m=0}^{\infty }\frac{{\left[-q_{0}(z)\right]}^{m}}{{\left(m+1\right)}^{3/2}}$$ (5)

or *q*_0_(*z*) < 1 [24],

where,

q0(z)=βI0Leff(1+z2zR2),

zR=kω022 = diffraction length of the beam

*ω*_0_ = beam waist radius at the focal point

*k* = 2*π*/*λ* = Wave vector.

Then, the nonlinear absorption coefficient, *β*, is given by [25]

$$\beta =\frac{2\sqrt{2\Delta T_{p-v}}}{l_{0}L_{eff}}$$ (6)

The real and imaginary parts of the third-order NLO susceptibility, *χ*^*(3)*^, were evaluated using the *n*_2_ and *β*, respectively. The relationships [26] are defined as follows:

$$Re\;\chi^{\left(3\right)}(esu)=10^{-4}\frac{\varepsilon_{0}c^{2}n_{0}{}^{2}}{\pi }n_{2}\;(\frac{cm^{2}}{W})$$ (7)

and

$$lm\;\chi^{\left(3\right)}(esu)=10^{-2}\frac{\varepsilon_{0}c^{2}n_{0}{}^{2}\lambda }{4\pi^{2}}\beta \;(\frac{cm^{2}}{W})$$ (8)

where,

*ε*_0_ = Vacuum permittivity,

*c* = Light velocity in vacuum.

The following relation gives the absolute value of the third-order NLO susceptibility [26]:

$$|\chi^{3}|={\left[{\left(R_{e}(\chi^{3})\right)}^{2}+{\left(I_{m}(\chi^{3})\right)}^{2}\right]}^{1/2}$$ (9)

### Theoretical DFT calculation

The starting geometries of the compounds were obtained using X-ray refinement data. The optimization of the molecular geometries, leading to energy minima, was achieved using DFT [with Becke’s non-local three-parameter exchange and the Lee-Yang-Parr correlation functional (B3LYP)] with the 6–311++G(d,p) basis set, as implemented in GAUSSIAN 09 program package [27]. The potential energy surface (PES) of **Anth-1** and **Anth-2** was scanned using B3LYP/6-311G++ (d,p) of the selected dihedral angle with an increment of 10° to localize the structure corresponding to the energy minima. The vibrational wavenumber and isotropic chemical shifts were calculated using the optimized structural parameters. The calculated vibrational frequencies were uniformly scaled-down with a scaling factor of 1.0065 (**Anth-1**) and 0.9971 (**Anth-2**) for frequencies less than 1700 cm^-1^, and 0.9948 (**Anth-1**) and 0.996 (**Anth-2**) for higher frequencies [28]. The ^1^H and ^13^C NMR chemical shifts in ppm were calculated relative to TMS as an internal standard using the gauge-invariant atomic orbital (GIAO) method, which computes the absolute chemical shielding owing to the electronic environment of the individual nuclei and is more accurate than other approaches for the same basis set [29]. The HOMO–LUMO energies, oscillator strengths, and absorption wavelengths, λ_max_, were calculated using time-dependent density functional theory (TD-DFT) at 6–311++G (d, p) and compared with the experimental UV absorption spectra. Furthermore, the molecular electrostatic potential (MEP) and Mulliken and ground-state dipole moments were computed using the same level of theory (B3LYP/6-311++G(d,p) basis set). Natural bond orbital (NBO) analysis was performed using the NBO 3.1 program implemented in the Gaussian 09 package.

### Hirshfeld surface analysis

The Hirshfeld surface and the related 2-D fingerprint plots for **the Anth-1** and **Anth-2** crystal structures were obtained using Crystal Explorer 3.1 [30]. The Hirshfeld surface, used to study the close interactions in the crystals, is shown as a 3-D image and a 2-D fingerprint plot; the surface enables a graphical understanding of the intermolecular interactions. The distances between the surface and the nearest atom inside and outside the surface are represented as *d*_*i*_ and *d*_*e*_, respectively [31, 32]. The normalized contact distance (*d*_*norm*_), calculated using Eq **10** based on the *d*_*i*_, *d*_*e*_, and van der Waals radii of the corresponding atom, provides a color-coded system for identifying intermolecular interactions. The blue color refers to the low frequency of the (*d*_*i*_, *d*_*e*_) pair, and the gray color corresponds to the full fingerprint [33].

$$d_{norm}=\frac{d_{i}-r_{i}{}^{vdW}}{r_{i}{}^{vdW}}+\frac{d_{e}-r_{e}{}^{vdW}}{r_{e}{}^{vdW}}$$ (10)

Where *d*_*i*_ is distance to the nearest nucleus internal to the surface and *d*_*e*_ is distance from the point to the nearest nucleus external to the surface. *r*_*i*_^*vdw*^ and *r*_*e*_^*vdw*^ are the van der Waals (vdW) radii of the internal and external to the surface, respectively.

## Results and discussion

### Molecular and optimized structural analyses

**Fig 4:**
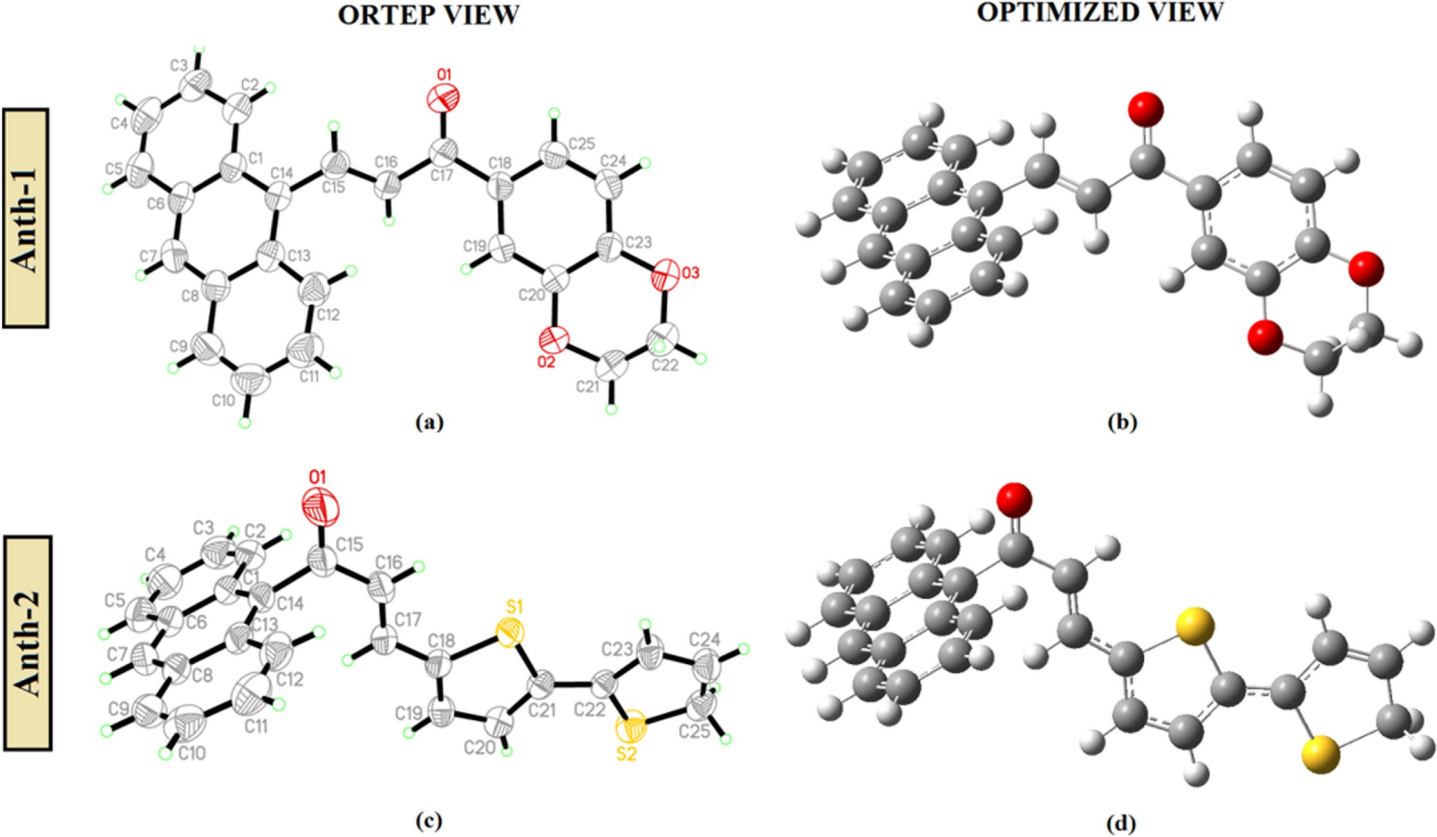
The molecular structure and optimized structure.

X-ray crystallographic analysis is effective for correlating molecular structures with their molecular properties. **Anth-1** and **Anth-2** were synthesized with different scaffolding positions of donor anthracene groups of 9-anthracenecarboxaldehyde and 9-acetylanthracene systems, respectively. These anthracene systems were attached to different substituents of 1,4-benzodioxan-6-yl methyl ketone and 2,2-bithiophene-5-carboxaldehyde, which act as electron-donating moieties. The ORTEP and optimized structures of both compounds are shown in Fig 4, and the crystal structures of **Anth-1** and **Anth-2** belong to the monoclinic and orthorhombic systems with space groups *P*2_1_ and *Pbca*, respectively. Additionally, the optimization process using DFT at the B3LYP/6-311G++(d,p) basis set without any constraints indicated a similarity between the selected geometrical parameters and the crystallographic data, as presented in S1 Table in S1 File. The bond distances and angles of both compounds were mutually consistent and within the normal ranges.

**Anth-1** stabilizes in the *s*-*cis* configuration with respect to the carbonyl group (C = O) [bond distance: 1.22 (3) Å; 1.23 (DFT) Å] and carbon-carbon double bond (C = C) [bond distance: 1.32 (4) Å; 1.34 (DFT) Å], while **Anth-2** adopts the *s*-*trans* conformation with respect to the carbonyl group (C = O) [bond distance: 1.22 (2) Å; 1.23 (DFT) Å] and carbon-carbon double bond (C = C) [bond distance: 1.45(3) Å; 1.46 (DFT) Å]. The short C–C distances in the central ring of the anthracene units for both AC are similar to other rings (C2–C3, C4–C5, C9–C10, and C11–C12) and consistent with the electronic structure of the anthracene units, where a central ring displaying aromatic delocalization is flanked by two isolated diene units [34].

**Fig 5:**
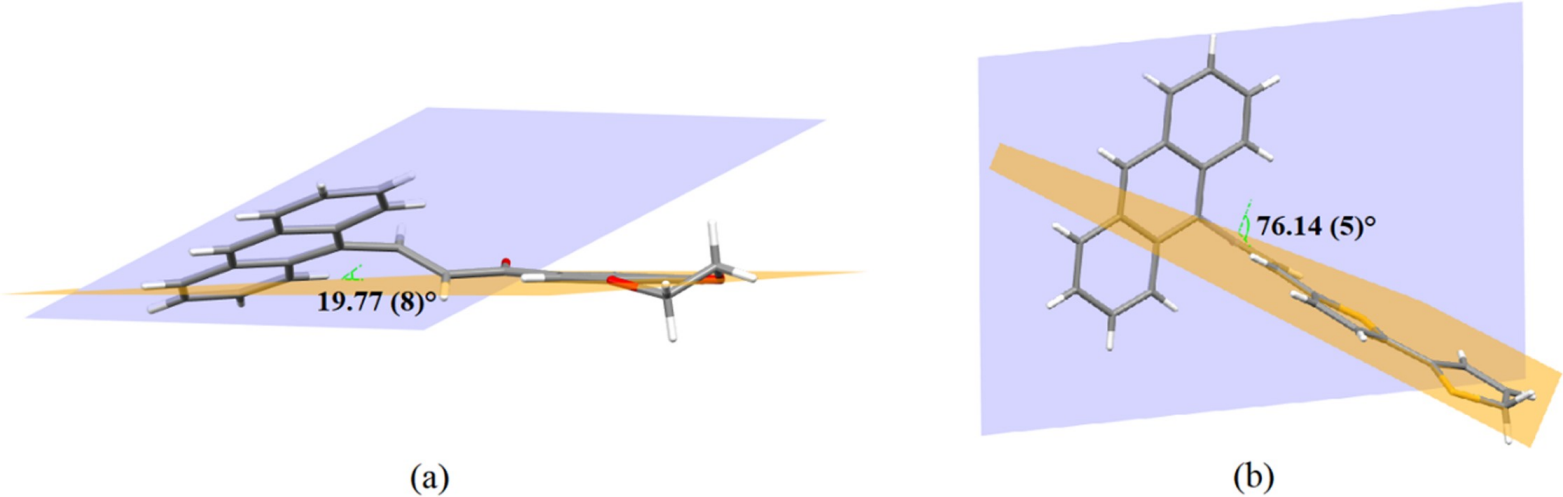
Dihedral angle between two planes. (a) **Anth-1**. (b) **Anth-2**.

The anthracene ring system (C1–C14) is slightly twisted at the (C15–C16) bond due to the enone moiety [C15–C17/O1, with a maximum deviation of 0.104 (3) Å for atom C17] with a C14–C15–C16–C17 torsion angle of –175.9 (2)° for **Anth-1**. However, a large distortion was observed in **Anth-2** between the anthracene and the enone moiety [C15–C17/O1; maximum deviation 0.036 (2) Å for atoms O1 and C16] at (C14–C15) bond with C1–C14–C15–O1 torsion angle of 90.8 (3)°. Both compounds possess a non-planar structure, where the plane of the anthracene moiety forms a dihedral angle of 19.77 (8)° with the 2,3-dihydrobenzo[*b*][1,4]dioxine plane (**Anth-1**) and 76.14 (5)° with the 2,2’-bithiophene plane (**Anth-2**), as shown in Fig 5A and 5B, respectively. The large dihedral angle observed in **Anth-2** suppresses the conjugation effect reducing the electronic effect between the two plane systems [35, 36]. The planarity of the molecule contributes to NLO behavior owing to the ease of charge transfer within the molecules, and the twisted molecules help to consolidate the crystal structure via intermolecular interactions.

**Fig 6:**
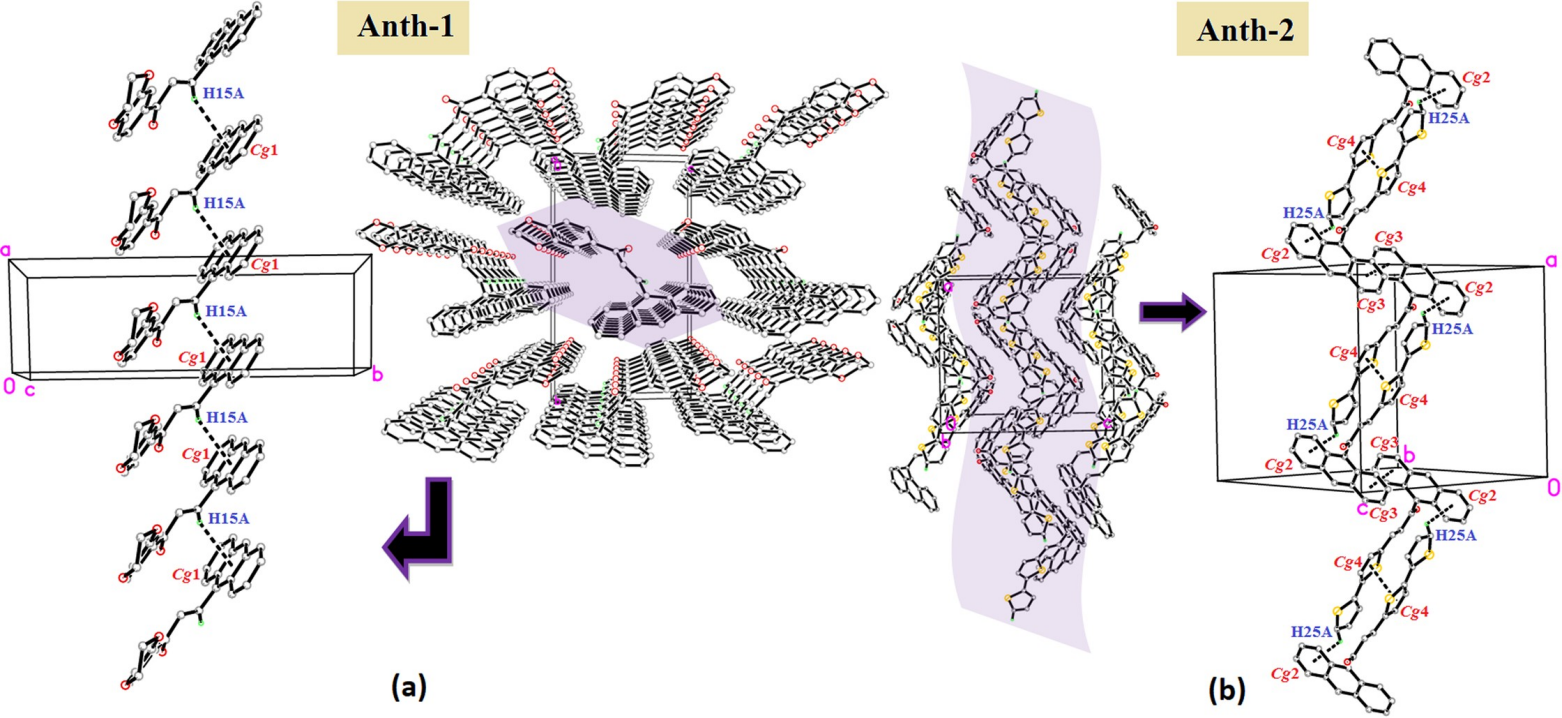
Weak C–H^…^π and π ^…^π interactions. (a) **Anth-1**. (b) **Anth-2**.

In **Anth-1**, C–H···π (*Cg*1) interactions (Table 2) exist between the anthracene ring and the enone bridge with the value of C···*Cg* as 3.797(3) Å (Table 3), and *Cg*1 is the centroid of the ring C1/C6–C8/C13–C14. The interactions link the molecules in a head-to-tail arrangement into infinite zig-zag chains along the crystallographic *a*-axis (Fig 6A).

**Table 2:** Hydrogen bond geometry.

	D–H⋯A	D–H (Å)	H⋯A (Å)	D⋯A (Å)	D–H⋯A (°)
**Anth-1**	**C15–H15A⋯*Cg*1** ^**i**^	0.93	2.97	3.797(3)	148
**Anth-2**	**C25–H25A⋯C*g*2** ^**ii**^	0.93	2.78	3.492(2)	135

Symmetry code: (i) *x*-1, *y*, *z*; (ii)–*x*+3/2, -*y*+1, -*z*+1.

**Table 3:** Experimental and theoretical ^1^H and ^13^C isotropic chemical shifts (ppm).

	Chemical shifts, δ(ppm)			
	Anth-1		Anth-2	
	Exp.	DFT^a^	Exp.	DFT^a^
	^1^H NMR			
α	7.86 (*J* = 9.5 Hz)	7.82	7.56 (*J* = 9 Hz)	7.84
Β	8.16 (*J* = 9.5 Hz)	9.52	8.19 (*J* = 8 Hz)	9.40
Phenyl Ring	4.21–7.87	4.17–8.35	7.00–7.95	4.36–7.0
Anthracene Ring	6.86–9.04	7.73–8.92	7.12–8.77	7.76–8.58
	^13^C NMR			
C15 = O	189.18	189.84	198.89	206.30
α	122.79	135.11	125.76	130.79
Β	143.57	151.87	141.89	143.54
C-Aromatic	64.30–139.35	67.69–154.55	126.20–140.21	52.71–153.9

^a^The isotropic chemical shift with respect to tetramethylsilane (TMS) calculated in DFT/B3LYP/6-311++G(d,p), which are 31.9681 ppm for ^1^H NMR and 184.0053 ppm for ^13^C NMR.

The crystal packing in **Anth-2** is stabilized through weak C–H⋯π (*Cg*2) interactions (Table 2) with a D–A distance of 3.797(3) Å, and the crystal packing also exhibited weak π⋯π interactions involving *Cg*3⋯*Cg*3 = 3.7518(11) Å (symmetry code: −*x*+1, −*y*+2, −*z*+2] and *Cg*4⋯*Cg*4 = 3.6282(14) Å [symmetry code: −*x*+1, −*y*+2, −*z*+2]. *Cg*2, *Cg*3, and *Cg*4 are the centroids of rings C1–C6, C8–C13, and S1/C18–C21, respectively. These intermolecular C–H⋯π and π⋯π interactions link the molecules in a side-by-side arrangement into sheets parallel to the *ac plane* (Fig 6B). Substituting bithiophene in **Anth-2** facilitated increased interactions which further stabilized the crystal structure.

The molecules in the Anth-1 and Anth-2 were packed into one-and two-dimension due to the intermolecular C–H^…^π and π ^…^π interactions. The molecules are packed in a side-by-side or head-to-tail fashion, enhancing the intermolecular charge transfer between the donor and acceptor groups of the molecules [18]. These positions improve the charge-transfer process between the molecules and increase the nonlinear susceptibility (χ3).

### Potential energy surface

**Fig 7:**
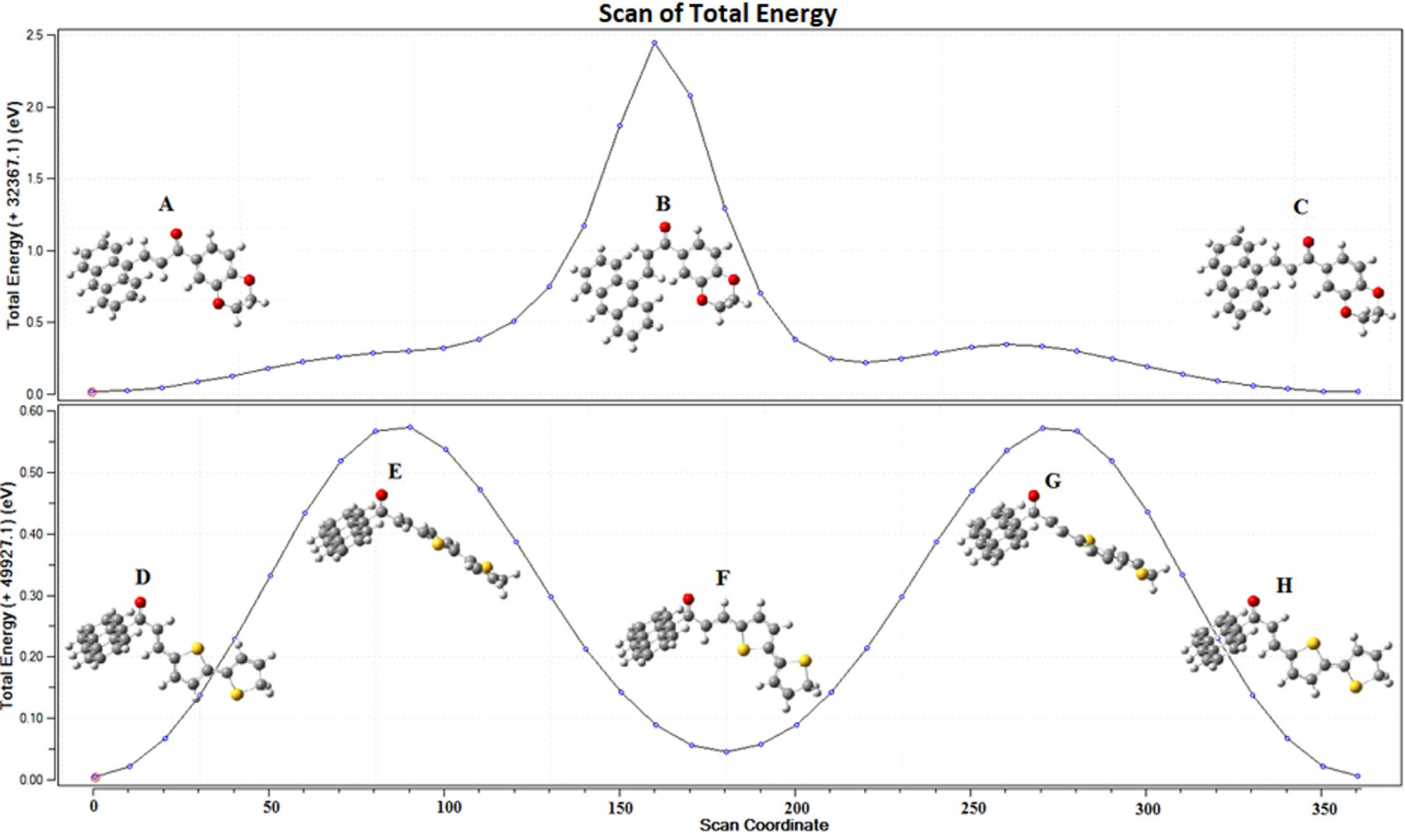
The potential energy curves.

PES determines the most stable conformation of a molecule through energy minimization. Energy-minimization calculations of the **Anth-1** and **Anth-2** structures were performed using DFT at the B3LYP/6-3111G++(d,p) implemented in the Gaussian program [27]. Fig 7 shows the PES of **Anth-1** and **Anth-2** for the O1–C17–C16–C15 (**Anth-1**) and O1–C15–C16–C17 (**Anth-2**) dihedral angle with an increment of 10°, which corresponds to the localized structure with minimum energy.

In **Anth-1**, conformation **B** has a maximum energy of 2.4434 eV, representing the *s-trans* configuration. The rest of the configurations exhibit minimum energies of 0.0175 (**A**) and 0.0179 (**C**) eV, representing the *s-cis* conformation. Contrastingly, **Anth-2** has three minimum energies representing the *s-trans* conformation with values of 0.0053 (**D**), 0.04582 (**F**), and 0.00591 (**H**) eV. The conformational energy profile has two maxima at 90° and 280° dihedral angles with energies of 0.5731 (**E**) and 0.5713 (**G**) eV, and indicates *s-cis* conformation with the inner hydrogens on C–14 and C–16 close to each other. This proximity leads to van der Waals repulsion making the *s-cis* conformation less stable than the *s-trans* conformation of **Anth-2** [37]. The most stable molecular structure of **Anth-1** and **Anth-2**, derived from PES, was comparable to the X-ray analysis, where **Anth-1** was stable in the *s-cis* conformer, and **Anth-2** was stable in the *s-trans* state.

### Hirshfeld surface analysis

**Fig 8:**
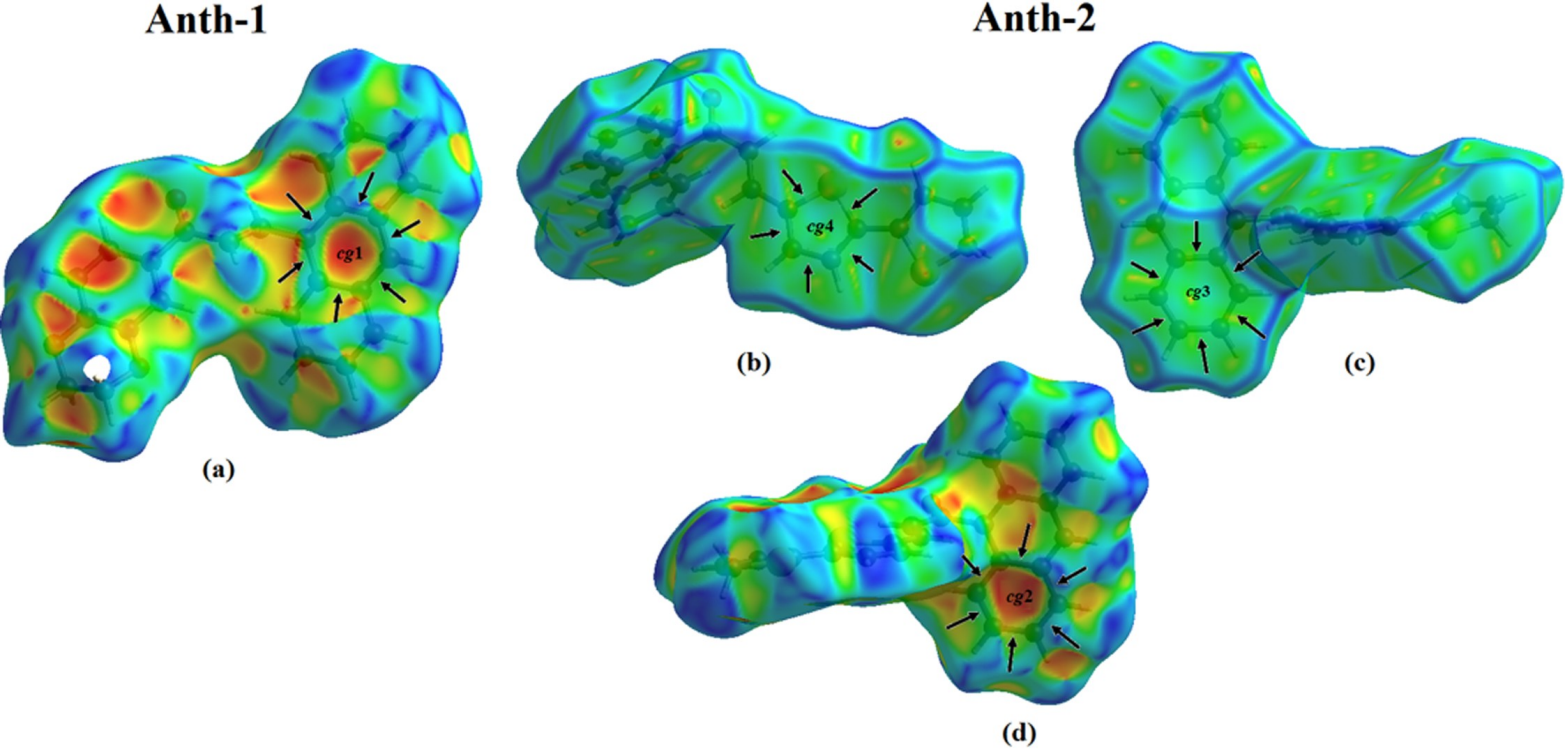
View of the Hirshfeld surface shape index and curvedness for Anth-1 and Anth-2.

The nature of the intermolecular contacts inside the unit cell packing was analyzed using Hirshfeld surface analysis, and the shape index [38], surface, and curvature are shown in Fig 8. The pale orange and bright red spots present on the shape index surface of **Anth-1** and **Anth-2** (identified with black arrows) confirm the presence of C–H⋯ π interactions (Fig 8A and 8D). The large, flat green curvature of the shape index surface of **Anth-2** (Fig 8B and 8C) confirms the presence of π ⋯ π interactions.

**Fig 9:**
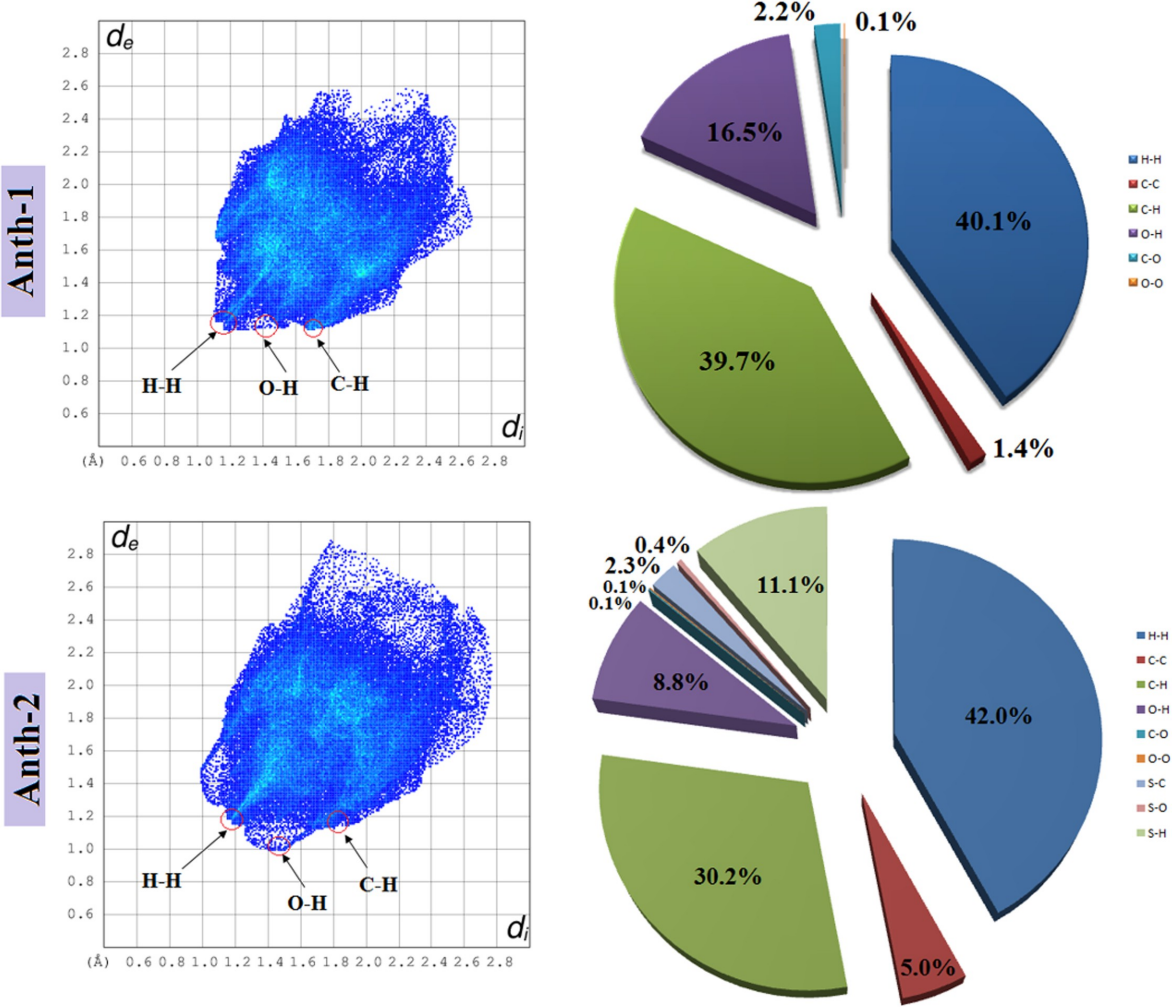
Fingerprint plots and the percentage of contacts full 2D fingerprint plots.

The results of the Hirshfeld surface analysis were analyzed using the two-dimensional fingerprint plots (Fig 9), indicating a difference between the intermolecular interaction patterns and the primary intermolecular contacts associated with both compounds. The H⋯H contact, as shown in Fig 9, is the major contributor, with a contact of 40.1% (**Anth-1**) and 42.0% (**Anth-2**), to the Hirshfeld surface, and is shown as a distinct spike with the minimum *d*_*e*_+*d*_*i*_ value less than the sum of the van der Waals radii (2.4 Å). The intermolecular C–H⋯π interactions for **Anth-1** and **Anth-2** have small interatomic C⋯H/H⋯C contact percentage of 39.7% (**Anth**-1) and 30.2% (**Anth-2**), showing two distinct spikes with *d*_*e*_+*d*_*i*_~2.8 Å (**Anth**-**1**) and 3.0 Å (**Anth-2**). The π⋯π (C⋯C) interactions have a contribution of 1.4% and 5.0% to the total Hirshfeld surface of **Anth-1** and **Anth-2**, respectively, and appear as a significant spike in the middle of the 2-D fingerprint plot.

### Fourier transform infrared spectroscopy analysis

**Fig 10:**
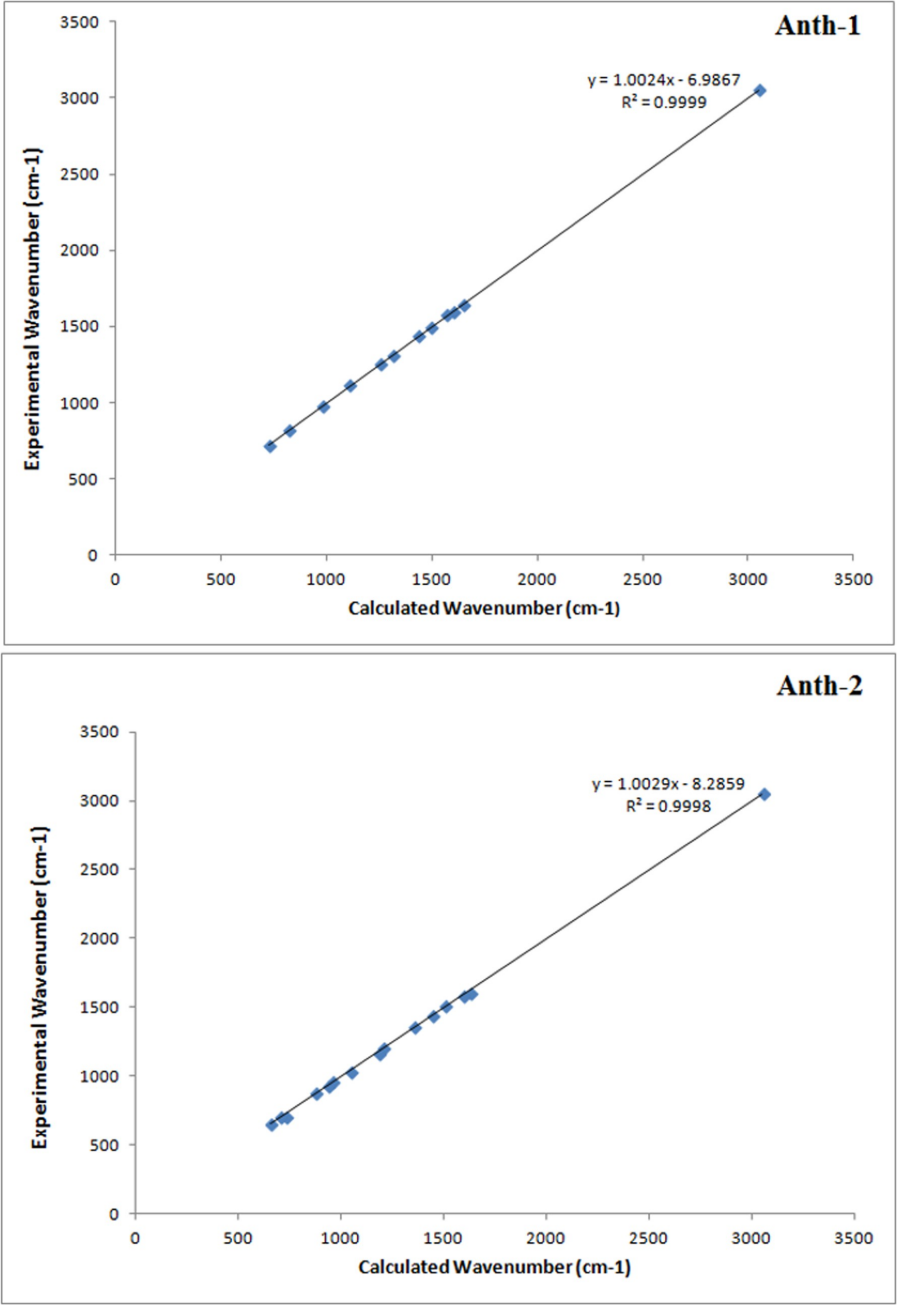
Correlation graph between the calculated and experimental vibrational frequencies.

The chemical bonding and molecular structure of the compounds were studied using infrared spectral analysis. The experimental and predicted infrared spectra are given in S1 and S2 Figs in S1 File, respectively. S2 Table in S1 File contains few theoretical and experimental vibrational frequencies, along with their assignments. A correlation graph between the calculated and experimental vibrational frequencies is shown in Fig 10, which indicates a good correlation between the theoretical and experimental vibrational frequencies with a high correlation coefficient [R^2^  =  0.9999 (**Anth-1**) and 0.9998 (**Anth-2**)].

The C–H stretching mode is observed above 3000 cm^−1^ as a multiple of weak to moderate bands compared to the aliphatic C–H stretch [39]. The absorption bands for **Anth-1** and **Anth-2** at 3051 and 3049 cm^−1^, respectively correspond to the C–H aromatic stretching vibrations. The theoretical DFT also aligns well with the experimental results, and the wavenumbers for C–H stretching frequencies are in the range of 3000–3125 cm^−1^ [40].

Carbonyl stretching C = O vibration is expected in the region of 1600–1700 cm^−1^ [41]. The experimental C = O stretching vibrations for **Anth-1** and **Anth-2,** observed at 1655 and 1625 cm^−1^, respectively, align well with the theoretically reported values of 1645 and 1606 cm^−1^ for **Anth-1** and **Anth-2**, respectively. Additionally, a strong band was observed because of the significant dipole moment owing to the large partial positive and negative charge of the carbonyl carbon and oxygen, respectively.

Owing to the aromatic ring vibrations, a strong C = C band of the compounds was observed between 1572 (**Anth-1**) and 1592 (**Anth-2**) cm^−1^, comparable to the theoretical values of 1581 (**Anth-1**) and 1588 (**Anth-2**) cm^−1^. The vibrations are sensitive to the amount of charge transfer between the donor and acceptor groups, leading to the formation of a heavy doublet, and these results compare well with earlier reports [42, 43]. According to Socrates (1981), the C = C stretching mode is expected around 1600 cm^−1^ when conjugated with the C = O group. The anthracene ring C = C stretching modes are expected between 1156–1615 cm^–1^ [44]. We observed the experimental and theoretical anthracene stretching between 1082–1665 cm^–1^ and 727–1655 cm^–1^, respectively. The activation of the C = C anthracene stretching mode confirms the charge transfer between the C = O group and the anthracene fused ring via the ethylenic bridge.

### Nuclear magnetic resonance analysis

The sample purity and structural conformation were examined using ^1^H and ^13^C NMR, and the observed characteristic peaks are listed in Table 5. For the two AC compounds (Scheme 1), the H-*α* and H-*β* protons resonated as two doublets in the ^1^H NMR spectra in the region of 7.86 (**Anth-1**), 7.56 (**Anth-2**) and 8.16 (**Anth-1**), 8.19 (**Anth-2**) ppm, respectively. According to DFT calculations, H-*α* and H-*β* protons resonated in the range of 7.82 (**Anth-1**), 7.84 (**Anth-2**) and 9.52 (**Anth-1**), 9.40 (**Anth-2**) ppm, respectively. The strong coupling constant indicates the *trans* coupling of the adjacent protons and confirms the *trans* configuration of the molecules for the C15–C16 (**Anth-1**) and C16–C17 (**Anth-2**) double bonds [45]. The proton chemical shifts, integrating for 9 protons, of the anthracene ring for **Anth-1** and **Anth-2** are observed in the de-shielded area between δ = 6.86–9.04 and 7.12–8.77 ppm, respectively, while the theoretical DFT values range between 7.73–8.92 (**Anth-1**) and 7.76–8.58 (**Anth-2**) ppm. The anthracene moiety of both compounds can be confirmed from the increased downfield chemical shifts between 7–9 ppm. The aromatic substituent protons were in the normal range. The δ values of the ^1^H NMR depend on the type of aromatic ring and vary with the electronic effects of the substituent on the ring.

In ^13^C NMR spectra, the carbonyl carbon appears at the most deshielded δ values of 189.18 (**Anth-1**) and 198.89 (**Anth-2**) ppm, while theoretically, it is located at 189.84 (**Anth-1**) and 206.30 (**Anth-2**) ppm. Experimentally, *α*- and *β*- carbon atoms exhibit characteristic signal at 122.79 (**Anth-1**) and 141.89 (**Anth-2**), and 143.57 (**Anth-1**) and 141.89 (**Anth-2**) ppm, respectively, while from DFT, it is calculated at 135.11 (**Anth-1**), 130.79 (**Anth-2**) for *α*-carbon and 151.87 (**Anth-1**) and 143.54 (**Anth-2**) ppm for *β*-carbon. The remaining chemical shifts of the carbon atoms of the aromatic rings were within normal ranges (Table 3).

### UV-vis absorption analysis

**Fig 11:**
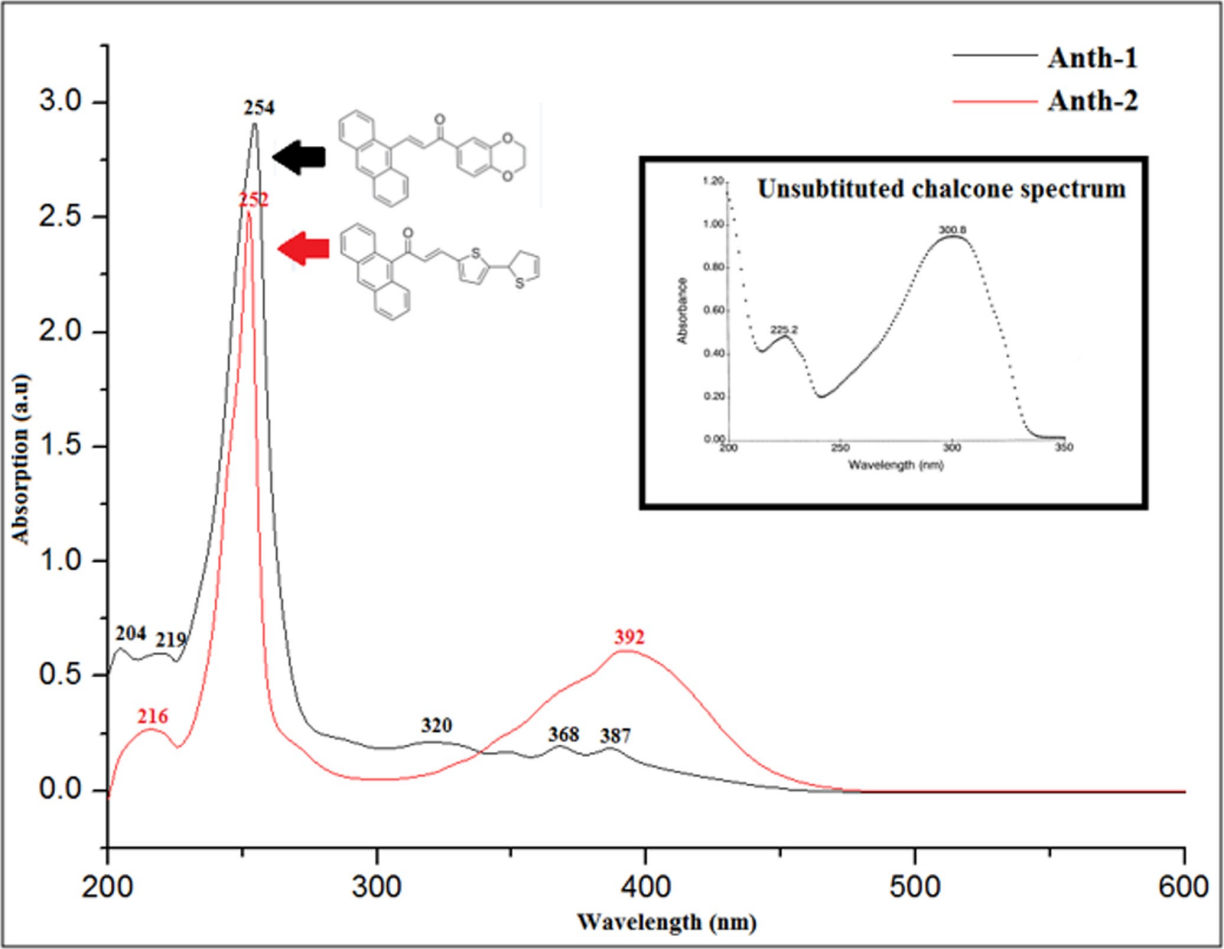
Experimental UV-Vis absorption spectra of Anth-1 and Anth-2.

The linear optical properties were analyzed using the UV-NIR spectrum. The TD-DFT at 6–311++G(d,p) was used to calculate the absorption wavelength (λ_max_) and HOMO–LUMO energies of the electronic transitions, which was compared with the experimental UV absorption spectra. The experimental and theoretical studies used the same solvent, DMSO. The influence of highly conjugated substituents at ring **A** and ring **B** (Scheme 1) on the absorption band is discussed further, and Table 4 summarizes the experimental and theoretical effect of the substituent in DMSO on the λ_max_ of **Anth-1** and **Anth-2** using unsubstituted chalcone as the reference. The substitution of the electron-donating group increases the λ_max_ values compared to the unsubstituted chalcone (Fig 11), while the substitution of phenyl in Ring **A** causes a bathochromic shift of λ_max_ in increasing order of conjugating power [46].

**Table 4:** Assigning observed and theoretical electronic transitions.

State	B3LYP/6-311++G(d,p)									
	Anth-1					Anth-2				
	λ calc. (nm)	Energy (eV)	*F*	State transition	λ Exp. (nm)	λ calc. (nm)	Energy (eV)	*F*	State transition	λ Exp. (nm)
1	380	2.71	0.2617	H→L	387	389	2.77	0.0001	H→L	392
				H→L+1					H→L+2	
2	-	-	-	-		389	2.77	0.0252	H-1→L	
3	-	-	-	-		390	2.79	0.0001	H→L+1	

The results in Table 4 summarize that **Anth-2** has the highest absorption maximum wavelength. In the experimental spectra, the strong absorption maximum *(λ*_max_) at 387 (**Anth-1**) and 392 (**Anth-2**) nm, corresponding to the π–π* transition, can be attributed to the excitation of the aromatic ring and C = O group [47]. The theoretical data of the electronic transition energies and the measured UV-vis data are presented in Table 4. The theoretical calculations are confined to the gaseous equivalent state, whereas the experimental data correspond to the solution state, resulting in a variation of the theoretical λ_max_ from the experimental results.

Comparing the AC derivatives with the unsubstituted chalcone, a shift is observed in the peak wavelength towards the long-wavelength region, and the height of absorption peaks increases with an increase in the conjugated system at Ring **A**. There is a two-fold increase in the bathochromic effect when the larger conjugated substituent is located on the phenyl ring **A** than on ring **B**. This may be attributed to the charge transfer in **Anth-2** [48] when the anthracene (strong donor and highly conjugated) ring is located near the carbonyl group (strong electron-withdrawing). Weakly positive groups, such as dioxine (**Anth-1**) and biothiophene (**Anth-2**), exhibit minor bathochromic effects when present on ring **B** and are absent on Ring **A** [48].

Substituting the electron-donating groups reduces the energy gap [49]. The energy band of **Anth-1** and **Anth-2** were calculated as 2.93 and 2.76 eV, respectively, by extrapolating the linear trend in the optical spectra. The low bandgap, on account of fused rings within the structures [50], makes these compounds suitable for optoelectronic applications [51].

### Frontier molecular orbitals and global chemical reactivity

**Fig 12:**
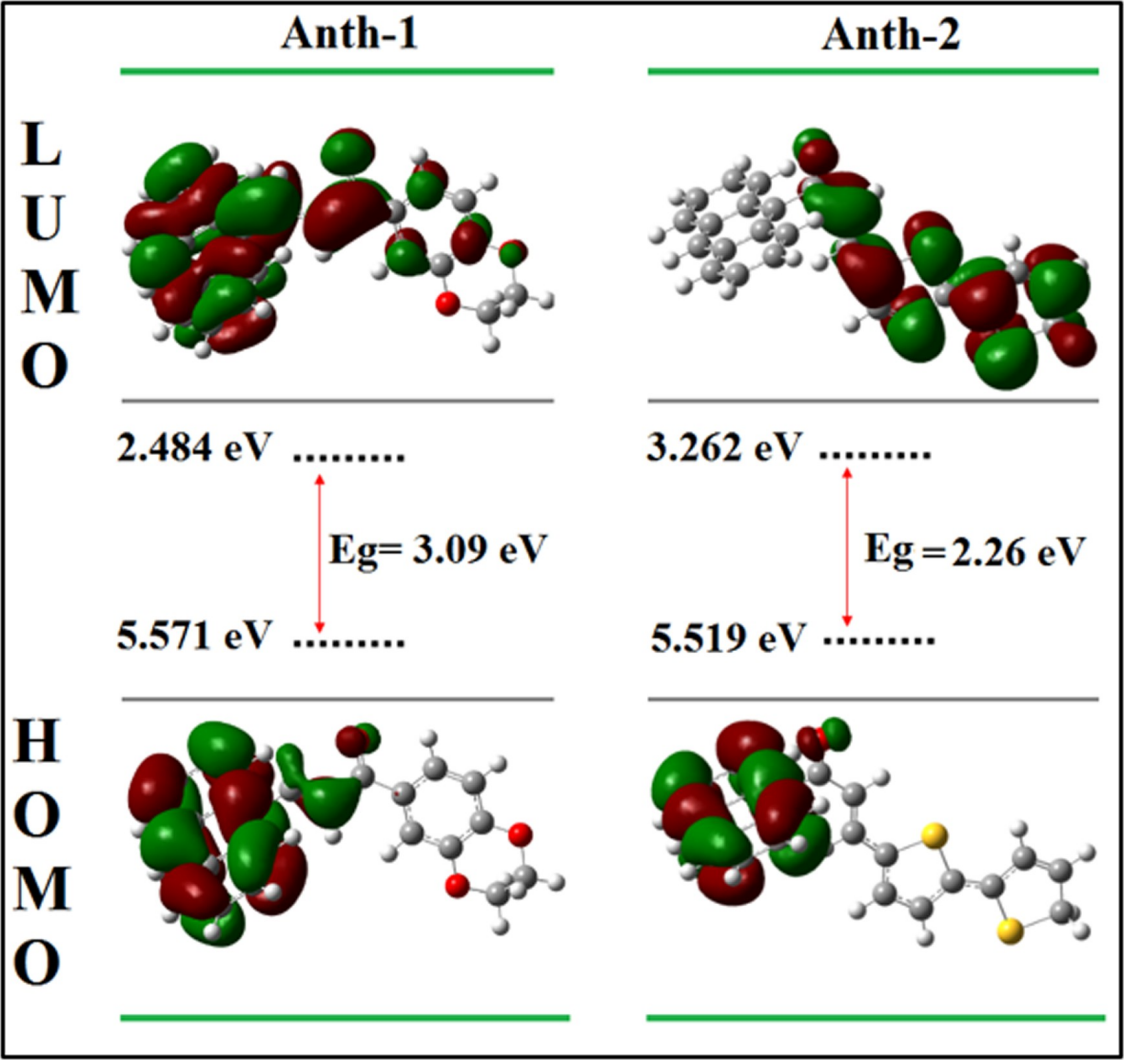
The frontier molecular orbitals.

The bonding scheme of the compounds can be explained using the surface of the frontier orbital, shown in Fig 12. The HOMO–LUMO energy gaps for **Anth-1** and **Anth-2** chalcones were estimated using the B3LYP/6-311++G (d, p) level of theory (Table 5). The positive and negative phases are represented using red and green colors, respectively. The frontier molecular orbital (FMO) results indicated that the HOMO electrons are distributed on the ethylenic bridge and the anthracene ring (strong donor). However, at the LUMO level, the charge densities are spread over the entire molecule (including the ethylenic bridge, aromatic rings, and carbonyl functional group). The energy gap for **Anth-1** and **Anth-2** were calculated as 3.09 and 2.26 eV, respectively. The DFT method exaggerates the delocalization of the frontier orbital HOMO–LUMO due to the self-interaction error, resulting in significant deviation of the bandgap values from the experimental results. The low HOMO–LUMO energy gap in these compounds indicates stronger chemical reactivity and lower kinetic stability, increasing the polarizability and NLO activity [52]. Substituting the electron-donating group with fused rings within the structures reduces the energy gap [49, 50], rendering these compounds suitable for optoelectronic applications [51].

**Table 5:** Calculated reactivity indices of AC.

Molecular energy (eV)	Anth-1	Anth-2
E_HOMO_	−5.5707	−5.5187
E_LUMO_	−2.4841	−3.2615
Energy gap (*Δ*)	3.09	2.26
Ionization potential (*I*)	5.5707	5.5187
Electron affinity (*A*)	2.4841	3.2615
Global hardness (*ƞ*)	1.5433	1.1286
Chemical potential (*μ*)	−4.0274	−4.3901
Global Electrophilicity (*ω*)	5.2549	8.5384
Softness (*S*)	0.6479	0.8861
Electronegativity (χ)	6.8127	7.1495

The ionization potential (*I*), electron affinity (*A*), chemical potential (*μ*), Global hardness (*ƞ*), Global Electrophilicity (*ω*), Softness (S), and Electronegativity (χ) were defined based on DFT. The *I* and *A* can be expressed using the HOMO and LUMO orbital energies as *I* = –E_HOMO_ and *A* = –E_LUMO_, respectively. The HOMO–LUMO energy gap reflects the chemical activity of the molecule, and a small energy gap is associated with specific intramolecular charge transfer from the donor to the acceptor via a π-conjugated path [53]. The energy gap determines the chemical reactivity, optical polarizability, and chemical hardness [54]. Soft molecules have small excitation energies enabling easy modification of electron densities, while it is difficult to change in hard molecules with large excitation energies [55]. The global hardness is given as *ƞ =* 1/2(ELUMO–EHOMO) ≈ ½ (*I*–*A*). The softness of a compound measures the extent of chemical reactivity, and it is the reciprocal of hardness, *S* = 1/*ƞ* [56]. **Anth-2** exhibits higher softness when it has lower energy gap than **Anth-1** (Table 5).

The escaping tendency of electrons in the molecules is given by electronic chemical potential (μ), which can be obtained using electron affinity and ionization energy [57], *μ =* 1/2(E_LUMO_ + E_HOMO_) ≈–½ (*I*+*A*). The electrophilicity index (*ω*) is a quantitative value intrinsic to a molecule used for predicting reactivity which was defined by Maynard [58] and Parr [59] defined ω as the tendency of an electrophile to acquire more electron density (chemical potential) divided by the resistance to exchange electron density with the environment (chemical hardness). The electrophilicity value is vital due to the enone moiety’s susceptibility to nucleophilic attack. While **Anth-2** was the softest, it contained the most electrophilic chalcones.

Absolute electronegativity, χ = (*I* + *A*/2), is related to the average value of the HOMO and LUMO energies defined by Mulliken [60]. Table 5 lists the reactivity indices of the compounds calculated using the TD-DFT method with the B3LYP/6-311++G(d,p) basis set in the ground state. Global reactivity descriptors establish **Anth-2** as a soft molecule (very high chemical reactivity and low kinetic stability) having higher intermolecular charge transfer.

### Mulliken and ground-state dipole moment

**Fig 13:**
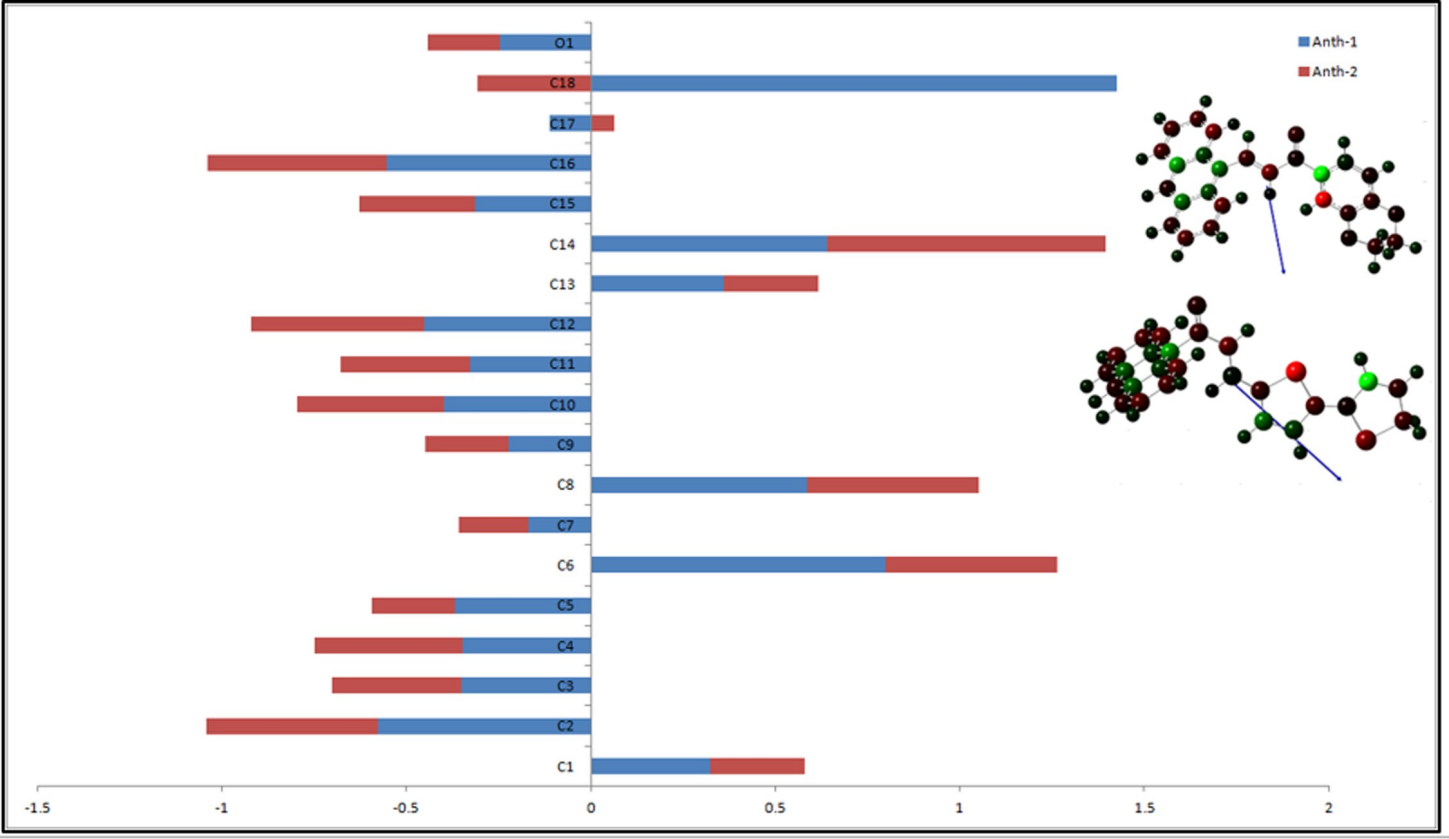
Orientation of electronic dipole moment vector (blue arrow).

The chemical bonds in the molecule depend on the Mulliken charges and regulate the dipole moment [61]. The total dipole moment and their components with charge-based colors of atoms are tabulated in Table 6 and shown in Fig 13. The analysis revealed a positive acceptor (electrophilic) and a negative donor (nucleophilic) atomic charge [62]. The magnitude of the carbon Mulliken charges range from –0.111 to 1.427 a.u. and –0.188 to 0.756 a.u. for **Anth-1** and **Anth-2**, respectively. Electrophilic charges are localized at all hydrogen atoms in the compound [62].

**Table 6:** The calculated dipole moments (Debye).

Code	μ_x_	μ_y_	μ_z_	μ_total_
**Anth-1**	-2.87	-1.57	4.09	5.24
**Anth-2**	-5.81	0.01	-3.47	6.77

The dipole vectors (Table 6) are indicated using arrows pointing along the bond towards the less electronegative atom, and the length of the arrow is proportional to the magnitude of the electronegativity difference between the atoms in each molecule. The dipole moment increased with an increase in the length of the conjugated system. **Anth-2** (6.77 D) exhibits a greater dipole moment than **Anth-1** (5.24 D) due to the higher net charge of the dipole moment and the difference in electronegativity between atoms. The increase in the dipole moment in **Anth-2** can also be attributed to the resonance effect of an electron-donating bithiophene substituent. Therefore, **Anth-2** has a better response towards NLO properties as it has a higher dipole moment than **Anth-1**.

### Molecular electrostatic potential

**Fig 14:**
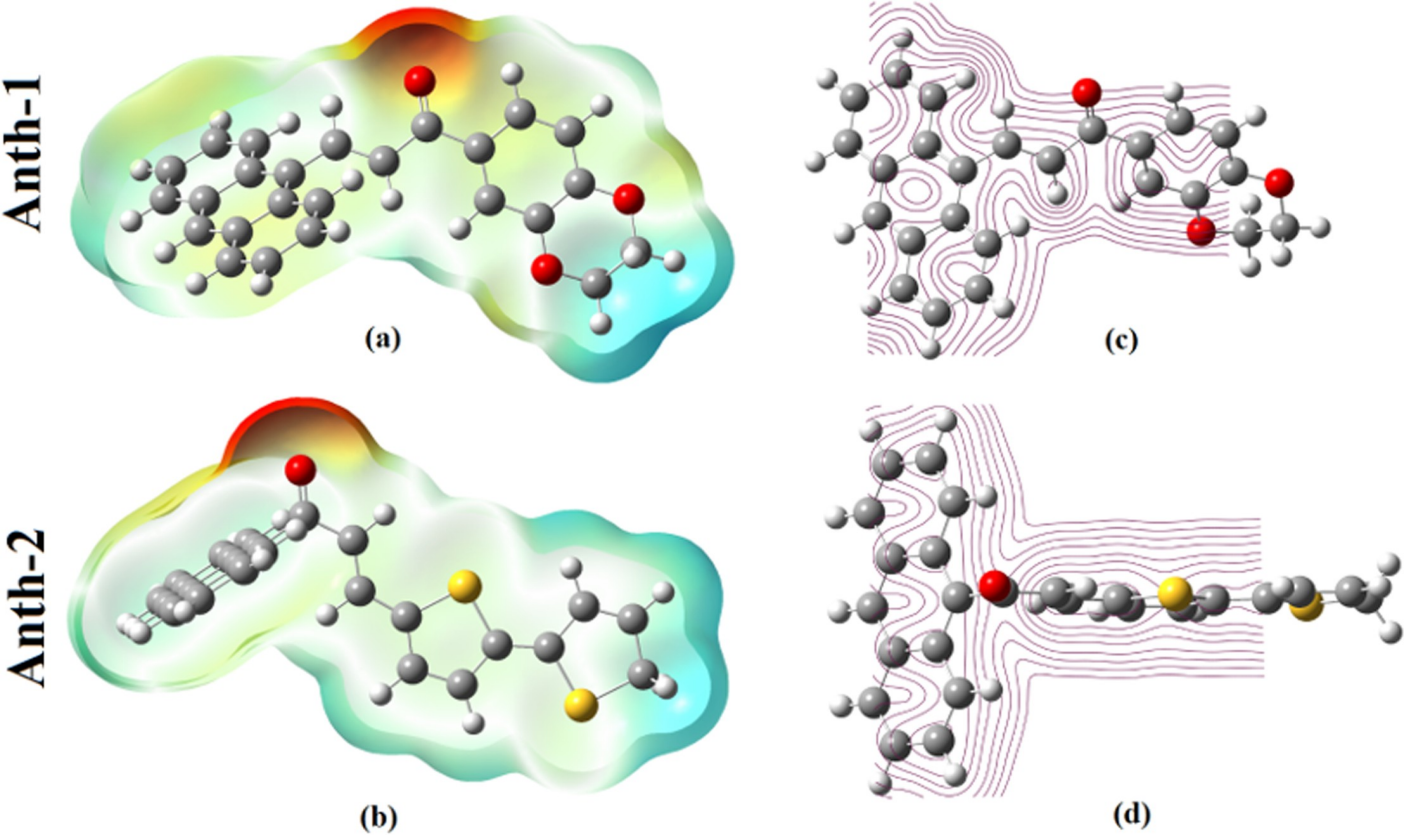
MEP plot of anthracenyl chalcones.

MEP corresponds to the distribution of electronic charges on the molecules and the shape of the molecules. MEP provides a visual method to understand the relative polarity of the molecules as it correlates with dipole moment, electronegativity, and partial charges. The electrostatic potential plot and contour map for the positive and negative potentials are shown in Fig 14. MEP indicates the regions susceptible to interaction with the environment and the preferred electrophilic or nucleophilic attack regions as well as the MEP map.

The color grading indicates molecular size, shape, and electrostatic potential value, helpful in studying the molecular structure and its physiochemical property [38]. The color scheme for the MEP surface includes red (electron-rich or partially negative charge), blue (electron-deficient or partially positive charge), light blue (slightly electron-deficient region), yellow (slightly electron-rich region), and green (zero potential region) [63].

The areas of low potential (red) contain abundant electrons and are localized over the oxygen atom, O, of the carbonyl group, which is the site for electrophilic attack. Contrastingly, the region with the maximum positive charge is the preferred site for the nucleophilic attack and is indicated by the blue color. The proton-rich region exhibits a positive potential and repulsion energy between the protons, which corresponds to the white region, and the carbon and hydrogen of green benzene correspond to an intermediate potential system.

The MEP contour plot is a two-dimensional map representing the charge distribution and displays the regions where the relative electron density falls within a specific range at (0010) for **Anth-1** and **Anth-2**. The electrostatic potential contour maps for the positive and negative potentials are shown in Fig 14C and 14D, respectively. The negative region was observed around the carbon atoms and oxygen, while the positive regions were near the hydrogen atoms.

### Natural bond orbital analysis

NBO analysis was performed by examining all possible interactions (hydrogen bond, intra-and intermolecular charge transfer) between the donor (Lewis-type) NBOs and acceptor (non-Lewis-type) NBOs, and estimating their second-order perturbation theory. The larger the energy of hyperconjugative interactions (E^(2)^), the more intensive the interaction between electron donors and electron acceptors; that is, the extent of conjugation of the system increases with an increase in the donating tendency from electron donors to electron acceptors. The energy of delocalization, ΔE_ij_, is calculated as

$$E^{\left(2\right)}=\Delta E_{ij}=q_{i}\frac{F{\left(i,j\right)}^{2}}{\epsilon_{j}-\epsilon_{i}}$$ (11)

where E^(2)^ is the energy of hyperconjugative interactions, q_i_ is the occupancy of the donating (Lewis type) orbital, ε_i_ and ε_j_ are the energies of the donating and accepting orbitals, respectively, and Fij is the off-diagonal element of the Fock matrix in the NBO basis [64].

The high hyperconjugative interaction energies (Tables 7 and 8) indicate the ICT in **Anth-1** and **Anth-2**, respectively, which induces NLO properties of molecular systems. In **Anth-1**, the charge transfer interactions between π* (C13–C14) and σ* (C15–H15) indicate C15–H15A⋯ π1 hydrogen bonding with 277.62 kcal/mol energy. The interaction between LP O1 to σ* (C18–C19), LP O2 to σ* (C21–C22), and LP O3 to σ*(C21–C22) yields large E^(2)^ energy values of 221.10, 553.18, and 269.32 kcal/mol, respectively, owing to the presence of an electron-donating oxygen atom attached to **Anth-1** [65].

**Table 7:** Second-order pertubation analysis of Fock-matrix in NBO analysis of Anth-1.

Donor (*i*)	Acceptor (*j*)	E^(2)^ kcal/mol	E(*j*)-E(i) a.u	F(*i*,*j*) a.u
σ(O2-C20)	σ*(C21-C22)	181.75	0.74	0.329
σ(O2-C21)	σ*(C21-H21)	36.30	2.80	0.285
	σ*(C21-C22)	639.23	0.10	0.232
σ(O3-C23)	σ*(C22-H22)	108.35	0.07	0.076
σ(C15-H15)	σ*(C18-C19)	116.19	0.07	0.079
LP 01	LP* C1	24.88	0.33	0.14
	σ*(C18-C19)	221.10	0.23	0.203
LP02	LP* C1	109.41	0.74	0.353
	σ*(C21-H21)	100.89	2.94	0.489
	σ*(C21-C22)	553.18	0.79	0.593
LP O3	σ*(C21-C22)	269.32	0.04	0.097
π*(C13-C14)	σ*(C15-H15)	277.62	1.81	0.491

**Table 8:** Second-order pertubation analysis of Fock-matrix in NBO analysis of Anth-2.

Donor (*i*)	Acceptor (*j*)	E^(2)^ kcal/mol	E(*j*)-E(i) a.u	F(*i*,*j*) a.u
σ(C1-C6)	σ*(C8-C13)	260.53	0.24	0.225
σ(C1-C14)	LP* (1) C13	44.10	0.18	0.095
σ(C4-H4)	σ*(C8-C13)	144.44	0.05	0.078
σ(C6-C7)	LP (1) C8	51.72	0.4	0.073
σ(C7-H7)	σ*(C8-C13)	141.38	0.04	0.069
σ(C25-H25)	σ*(C2-H2)	1280.83	6.60	2.596
	σ*(C1-C6)	35.51	7.90	0.489
	σ*(C3-C4)	1077.70	6.08	2.286
σ(S1-C21)	σ*(C20-C21)	157.36	1.26	0.399

In **Anth-2**, the charge transfer interactions between σ(C25–H25) and σ*(C2–H2), σ*(C1–C6) and σ*(C3–C4) provide evidence of C25–H25⋯ π2 hydrogen bonding with energies of 1280.83, 37.51 and 1077.70 kcal/mol, respectively. The interaction between π3 ⋯ π3 and π4 ⋯ π4 results in charge-transfer interaction from σ(C1–C6) to σ*(C8–C13) and σ(S1–C21) to σ*(C20–C21) with stabilization energy of 260.53 and 155.36 kcal/mol, respectively.

The results indicate that the ICT between the anthracene system and the substituent through an ethylenic bridge and the carbonyl group stabilizes the system, enhancing the NLO properties of **Anth-1** and **Anth-2**.

### Third order nonlinear optics

**Fig 15:**
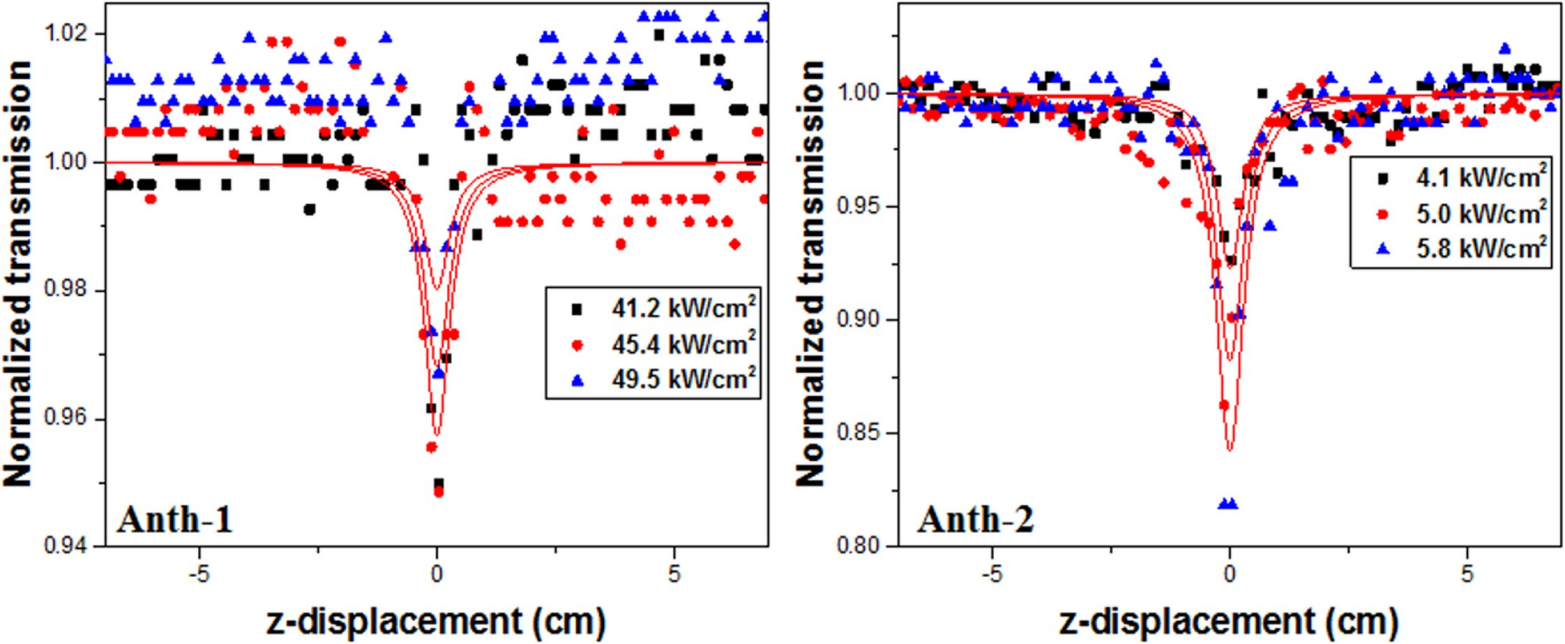
Normalized open aperture Z-scan.

**Fig 16:**
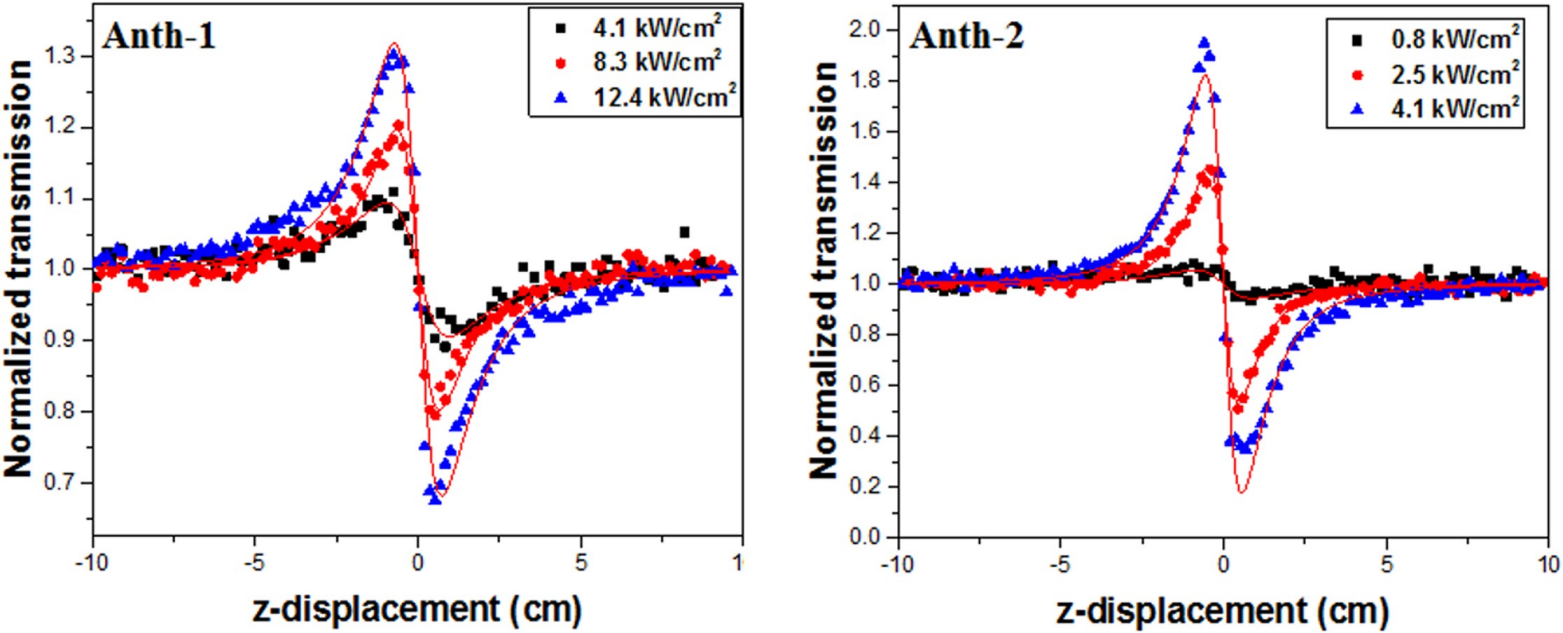
Normalized closed aperture Z-scan.

The Z-scan technique was used to measure the nonlinear refractive index and absorption coefficient for investigating the third-order NLO properties. The normalized open and closed aperture Z-scan traces of **Anth-1** and **Anth**-**2** are shown in Figs 15 and 16, respectively. The red lines represent the nonlinear fitting based on the Levenberg-Marquardt algorithm, and the corresponding values of *β* and *n*_*2*_ are listed in Tables 9 and 10, respectively.

**Table 9:** Open-aperture Z-scan parameters.

Anth-1			Anth-2		
Laser intensity (kW/cm^2^)	*β* (x10^-5^) (cm/W)	*Im* χ^3^ (x10^-6^) (esu)	Laser intensity (kW/cm^2^)	*β* (x10^-5^) (cm/W)	*Im* χ^3^ (x10^-6^) (esu)
41.2	2.30 ± 0.35	2.47	4.1	59.93 ± 4.09	64.33
45.4	2.86 ± 0.33	3.06	5.0	81.57 ± 4.77	87.55
49.5	1.16 ± 0.53	1.25	5.8	101 ± 4.87	108.41

**Table 10:** Closed-aperture Z-scan parameters.

Anth-1			Anth-2		
Laser intensity (kW/cm^2^)	*n*_*2*_ (x10^-9^) (cm^2^/W)	*Re χ*^*3*^ (x10^-6^) (esu)	Laser intensity (kW/cm^2^)	*n*_*2*_ (x10^-9^) (cm^2^/W)	*Re χ*^*3*^ (x10^-6^) (esu)
4.1	- 9.47 ± 0.37	2.40	0.8	- 27.12 ± 3.39	6.88
8.3	- 10.01 ± 0.23	2.54	2.5	- 78.87 ± 1.24	20.0
12.4	- 10.74 ± 0.24	2.72	4.1	- 83.22 ± 1.44	21.1

The open-aperture Z-scan measurement (Fig 15) was performed at 41.2, 45.4, and 49.5 KW/cm^2^ for **Anth-1** and 4.1, 5.0, and 5.8 KW/cm^2^ for **Anth-2.** The valley in the normalized transmittance for the open-aperture scan indicates a strong reverse saturation absorption at peak intensities. The shape of the open-aperture curves indicates the presence of two-photon absorption. When the sample is at the focal lens, maximum absorption occurs because the on-axial peak irradiance of the beam is highest at this point. The reduction in the transmittance for the open aperture is independent of the nonlinear refraction and can be used to determine the *β* using the depth of the valley. The value of *β* was found to be 2.30, 2.86, and 1.16 x10^-5^ cm/W and 4.1, 5.0, and 5.8 x10^-5^ cm/W for **Anth-1** and **Anth-2**, respectively, with different laser intensities.

Optical absorption causes heat transfer to the irradiated material during the light-matter interaction, potentially changing the properties of the material [66]. In this study, the absorption peaks were in the UV region, and the absence of linear absorption in the visible region indicates negligible thermal effects. The materials with reverse saturation absorption become opaque upon exposure to high photon fluxes due to the high absorption from the excited state [67]. **Anth-2** exhibited superior nonlinear absorption compared to **Anth-1** under continuous waves, enabling their optical limiting applications.

Both samples exhibited a self-defocusing effect with a negative nonlinear refractive index, tabulated against the focal intensities in Table 9. The transmission between the peak and valley (ΔT_P-V_) was calculated from the closed aperture Z-scan curves (Fig 16). The self-defocusing effect, on account of local variation in the refractive index with temperature, is essential for optical sensor applications, such as night vision devices [68], and increases the laser damage threshold [69].

Although the *n*_*2*_ should be independent of the intensity for pure third-order nonlinearity, the value of *n*_*2*_ in Fig 16 is marginally dependent on the intensity, indicating the presence of thermally induced nonlinearity. Therefore, the samples exhibit strong temperature-dependent nonlinearity.

**Anth-2** exhibited a superior third-order NLO response than **Anth-1**, and the higher imaginary χ^3^ compared to the real counterpart in **Anth-2** indicates the dominance of nonlinear absorption over nonlinear refraction. Therefore, **Anth-2** has significant potential for optical limiting applications. The absolute value of *χ*^*3*^ for **Anth-2** was calculated using the relation |*χ*^3^| = [(*Re*(*χ3*))^2^+(*Im*(*χ3*))^2^]^1/2^ at intensity of 4.1 kW/cm^2^, and was found to be 1.10 x10^-4^ esu. The value of *χ*^*3*^ cannot be calculated for **Anth-1** because different intensities are used for open and closed apertures. However, the values of *n*_*2*_ and *β* indicate that a lower third-order susceptibility than **Anth-2**. The *χ*^*3*^ of **Anth-2** is larger than some third-order NLO materials, such as organic polymers, organic metals, organic dyes, and Rh:BaTiO_3_ thin films, which yielded the values for *χ*^*3*^ in the order of 10^−7^ and 10^−8^ esu [70, 71].

## Conclusion

**Anth-1** and **Anth-2** were successfully synthesized and characterized using FTIR, ^1^H NMR, and ^13^C NMR techniques. **Anth-1** and **Anth-2** adopt *s-cis* and *s-trans* configurations at the enone bridge with respect to the C = O and C = C bonds, respectively. The *s-trans* conformation of **Anth-2** is thermodynamically stable because the *s-cis* conformation has a steric repulsion between the two hydrogens inside the anthracene ring and the other substituent ring. The molecules in the crystal are packed in two dimensions by intermolecular C–H⋯π and π⋯π interactions, and are positioned in a head-to-tail fashion side by side, enhancing the ICT between the donor and acceptor groups of the molecules. The higher maximum absorption of **Anth-2** (392 nm) is due to the significant charge transfer when the anthracene (strong donor and highly conjugated) ring is near the carbonyl group (strong electron-withdrawing). The absorption peak wavelengths shift toward longer wavelengths, resulting in a lower energy gap. The UV-Vis spectra revealed that the ICT of the two different structures influence the absorption peak shifts and the HOMO–LUMO energy levels. Both UV-Vis and FMO indicate a lower energy bandgap for **Anth-2**. Global reactivity descriptors indicate that **Anth-2** is soft (very high chemical reactivity and low kinetic stability) and has higher ICT. The dipole moment calculation shows that the electronegativity difference between the atoms leads to a strong push-pull effect and an increase in the dipole moment, resulting in an enhanced nonlinear response of organic molecules. The resonance effect of an electron-donating bithiophene substituent contributes to the higher dipole moment of **Anth-2**.

In MEP, the carbonyl group acts as a reactive site for electrophilic attack and the binding site for intermolecular C–H⋯O interactions, while the anthracene ring provides sites for nucleophilic attack. NBO analysis indicates that ICT in **Anth-1** and **Anth-2** induces the NLO properties of the molecules. The fast charge transfer between the strong donor (anthracene) and strong acceptor (carbonyl) group through the π-conjugation increases the nonlinearity of **Anth-2** because anthracene is located near the carbonyl group. The superior third-order NLO response of **Anth-2** is due to the molecular structure and the conjugated substituent factors. The higher magnitude of the imaginary χ^3^ in **Anth-2** indicates its potential use as an optical limiter.

The NLO properties of the materials can be attributed to the arrangement of the molecules in the crystal, strong conjugated electron donor substituents, small energy gap, and push-pull effect of the dipole moments. The enhanced electron affinities, extended conjugation lengths of molecular backbones, strong electron-donating substituents, and different positioning of the substituents within the molecule of D–π–A–D or D–A–π–D structure affect the structure-property relationship and, subsequently, the nonlinear properties of the compounds.

## Supporting information

S1 File Supporting information of figure, table and CheckCIF validation reports. (DOCX)

S1 Graphical abstract (TIF)
