# Supplementary material for: Fused ring effect on optical nonlinearity and structure property relationship of anthracenyl chalcone based push-pull chromophores
Source: PLoS One. 2021 Sep 28;16(9):e0257808. doi: 10.1371/journal.pone.0257808 (PMC8478194; doi:10.1371/journal.pone.0257808)
Supplement: S1 File — (DOCX) [file pone.0257808.s001.docx]

**Supporting information for:**

**Fused Ring Effect on Optical Nonlinearity and Structure Property Relationship of Anthracenyl Chalcone Based Push-Pull Chromophores**

Dian Alwani Zainuri^1¶^, Mundzir Abdullah^2¶^, Muhamad Fikri Zaini^1¶^, Hazri Bakhtiar^3¶^, Suhana Arshad^1¶^*, Ibrahim Abdul Razak^1¶^*

^1^X-ray Crystallography Unit, School of Physics, Universiti Sains Malaysia, 11800 USM, Penang, Malaysia.

^2^Institute of Nano Optoelectronics Research and Technology (INOR), Universiti Sains Malaysia, 11800 USM, Penang, Malaysia.

^3^Department of Physics, Faculty of Sciences, Universiti Teknologi Malaysia, Johor Bahru, Johor, Malaysia.

*Corresponding author

E-mail: arazaki@usm.my.

E-mail: suhanaarshad@usm.my.

^¶^These authors contributed equally to this work


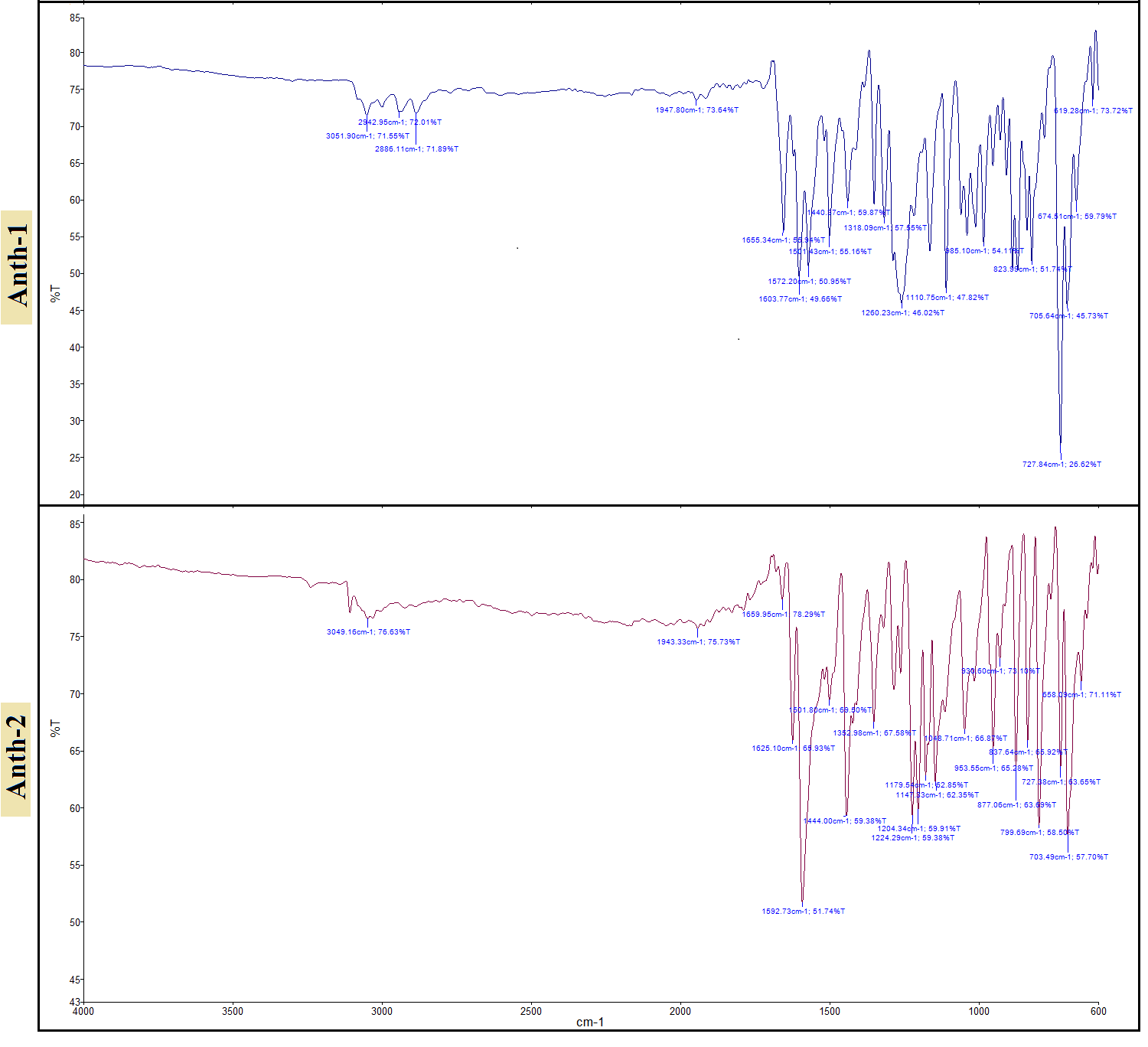


**S1 Fig. Experimental FTIR Spectra**.


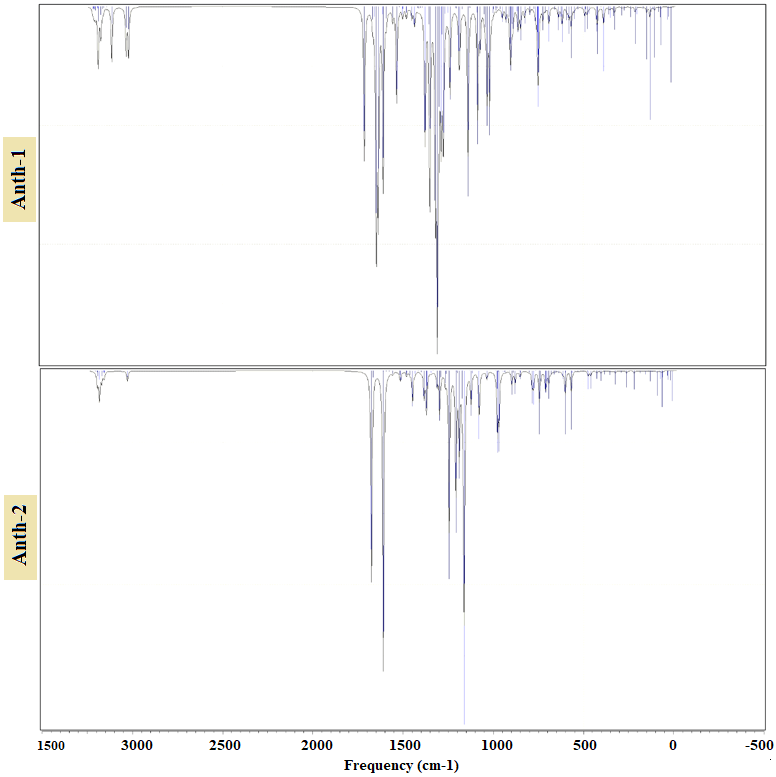


**S2 Fig.** **Theoretical FTIR Spectra**.

**S1 Table. Comparison between calculated (DFT) and X-ray values of selected geometrical data for Anth-1 and Anth-2.**

|  | **Anth-1** | |  | **Anth-2** | |
| --- | --- | --- | --- | --- | --- |
|  | **X-ray**  **(Å,^°^)** | **DFT (Å,^°^)** |  | **X-ray**  **(Å^°^)** | **DFT**  **(Å^°^)** |
| Bond Length |  |  |  |  |  |
| O1—C17/O1—C15 | 1.22(3) | 1.23 |  | 1.22(2) | 1.23 |
| C1—C14 | 1.42(3) | 1.42 |  | 1.41(3) | 1.41 |
| C1—C2 | 1.43(4) | 1.43 |  | 1.43(3) | 1.43 |
| C2—C3 | 1.36(4) | 1.37 |  | 1.36(4) | 1.37 |
| C3—C4 | 1.41(5) | 1.42 |  | 1.42(4) | 1.42 |
| C4—C5 | 1.35(5) | 1.37 |  | 1.35(4) | 1.37 |
| C5—C6 | 1.43(4) | 1.43 |  | 1.42(3) | 1.43 |
| C6—C7 | 1.39(4) | 1.40 |  | 1.39(3) | 1.40 |
| C7—C8 | 1.39(4) | 1.40 |  | 1.39(3) | 1.40 |
| C8—C9 | 1.43 (4) | 1.43 |  | 1.43 (3) | 1.43 |
| C9—C10 | 1.35 (4) | 1.36 |  | 1.36 (3) | 1.37 |
| C10—C11 | 1.40 (5) | 1.42 |  | 1.41 (4) | 1.42 |
| C11—C12 | 1.35 (4) | 1.37 |  | 1.36 (3) | 1.37 |
| C12—C13 | 1.43 (3) | 1.43 |  | 1.43 (3) | 1.43 |
| C13—C14 | 1.41 (3) | 1.42 |  | 1.40 (3) | 1.41 |
| C14—C15 | 1.48 (3) | 1.47 |  | 1.51 (4) | 1.51 |
| C15—C16 | 1.32(4) | 1.34 |  | 1.45(3) | 1.46 |
| C16—C17 | 1.49 (3) | 1.49 |  | 1.34 (3) | 1.36 |
| C17-C18 | 1.49 (2) | 1.49 |  | 1.44 (2) | 1.42 |
|  |  |  |  |  |  |
| Bond Angle |  |  |  |  |  |
| C14—C15—C16 | 125.3 (2) | 126.97 |  | 119.75 (16) | 119.19 |
| C15—C16—C17 | 122.1 (2) | 120.46 |  | 123.92 (17) | 124.31 |
| C16—C17—C18 | 119.2 (2) | 118.88 |  | 125.69 (17) | 127.14 |
|  |  |  |  |  |  |
| Torsion Angle |  |  |  |  |  |
| C1—C14—C15—C16 | -129.0 (3) | 131.69 |  | 98.1 (2) | -91.14 |
| C13—C14—C15—C16 | 51.0 (5) | 49.35 |  | -90.3 (2) | 91.13 |
| C14—C15—C16—C17 | -175.9 (2) | 179.32 |  | 8.5 (5) | -0.0013 |
| C15—C16—C17—C18 | 150.9 (3) | 172.16 |  | 176.22 (19) | 179.99 |
| C16—C17—C18—C19 | -6.3 (5) | 12.35 |  | -175.80 (19) | 179.99 |

**S2 Table. Experimental and theoretical vibrational frequencies of Anth-1 and Anth-2 and their tentative assignments.**

| **Anth-1** | | | **Anth-2** | | | IR  Assigments |
| --- | --- | --- | --- | --- | --- | --- |
| Exp.  (cm^-1^) | DFT | | Exp.  (cm^-1^) | DFT | |  |
|  | Unscaled (cm^-1^) | Scaled (cm^-1^) |  | Unscaled (cm^-1^) | Scaled (cm^-1^) |  |
| - | 3112 | 3132 | 3049 | 3058 | 3049 | *v_asym_*C–H |
| - | 3111 | 3131 | - | 3027 | 3018 | *v_asym_*C–H |
| 3051 | 3031 | 3051 | - | 1674 | 1667 | *v_asym_*C–H |
| - | 1665 | 1656 | 1625 | 1612 | 1606 | *v*C=C_anth_, ρCH_anth_ |
| 1655 | 1654 | 1645 | - | 1609 | 1603 | *v*C=C_anth_, ρCH_anth_ |
| - | 1644 | 1635 | 1592 | 1594 | 1588 | *v*C=C_phnyl_, ρCH_phnyl_ |
| 1603 | 1607 | 1599 | - | 1518 | 1512 | *v*C=C_anth_, ρCH_anth_ |
| 1572 | 1589 | 1581 | 1501 | 1513 | 1507 | *v*C=C_anth_, ρCH_anth_ |
| 1501 | 1499 | 1491 | 1444 | 1446 | 1440 | ρCH_anth_ |
| 1440 | 1447 | 1439 | - | 1400 | 1394 | ρCH |
| - | 1410 | 1403 | 1352 | 1368 | 1363 | *v*C=C_phnyl_, ρCH |
| 1318 | 1317 | 1310 | - | 1261 | 1256 | ρCH_anth,_ ρCH_phnyl_ |
| - | 1286 | 1279 | 1204 | 1207 | 1202 | ρCH_anth,_ ρCH_phnyl,_ ρCH |
| 1260 | 1263 | 1256 | - | 1188 | 1183 | *v*C=C_phnyl_, ρCH_phnyl_ |
| - | 1249 | 1243 | 1179 | 1172 | 1167 | δC=C_phnyl_ |
| - | 1136 | 1130 | 1048 | 1036 | 1032 | δC=C_anth_ |
| - | 1128 | 1122 | - | 1035 | 1031 | ωCH_anth_ |
| 1110 | 1122 | 1116 | - | 989 | 985 | *t*CH_anth_ |
| - | 1031 | 1026 | 953 | 967 | 963 | ρCH_anth,_ ρC=C_anth,_ *t*CH |
| - | 999 | 994 | 936 | 934 | 930 | δC=C_anth,_ *t*CH_anth,_ |
| - | 994 | 989 | - | 908 | 904 | δC=C_anth,_ *t*CH_anth,_ δC=C_phnyl_ |
| 985 | 983 | 978 | - | 897 | 893 | ωCH_anth_ |
| - | 975 | 970 | 877 | 878 | 874 | δC=C_phnyl_ |
| - | 856 | 852 | 727 | 711 | 708 | δC=C_anth,_ ρCH, *t*C=C_phnyl,_ *t*CH_phnyl_ |
| 823 | 823 | 819 | 703 | 704 | 701 | δC=C_anth,_ ρCH_anth,_ *t*CH, *t*C=C_phnyl,_ *t*CH_phnyl_ |
| - | 752 | 748 | 658 | 652 | 649 | δC=C_phnyl,_ ρCH |
| 727 | 721 | 717 | - | 600 | 598 | ωC=C_anth,_ δC=C_anth_ |

**S1 CheckCIF Validation Reports of Anth-1**

**checkCIF/PLATON (basic structural check)**

Structure factors have been supplied for datablock(s) mo_NAZ01_0m

THIS REPORT IS FOR GUIDANCE ONLY. IF USED AS PART OF A REVIEW PROCEDURE FOR PUBLICATION, IT SHOULD NOT REPLACE THE EXPERTISE OF AN EXPERIENCED CRYSTALLOGRAPHIC REFEREE.

No syntax errors found. [CIF dictionary](http://www.iucr.org/iucr-top/cif/cif_core/definitions/index.html)
Please wait while processing .... [Interpreting this report](http://journals.iucr.org/services/cif/checking/checkcifreport.html)

[Structure factor report](http://checkcif.iucr.org/0UOCcmBJqaSBV/031721140607147276000/ckf.html)

**Datablock: mo_NAZ01_0m**

| Bond precision: | C-C = 0.0041 A | Wavelength=0.71073 |
| --- | --- | --- |

| Cell: | a=5.4388(7) | b=17.080(2) | c=10.0539(12) |
| --- | --- | --- | --- |
|  | alpha=90 | beta=103.725(2) | gamma=90 |
| Temperature: | 296 K |  |  |

|  | Calculated | Reported |
| --- | --- | --- |
| Volume | 907.29(19) | 907.28(19) |
| Space group | P 21 | P 21 |
| Hall group | P 2yb | P 2yb |
| Moiety formula | C25 H18 O3 | C25 H18 O3 |
| Sum formula | C25 H18 O3 | C25 H18 O3 |
| Mr | 366.39 | 366.39 |
| Dx,g cm-3 | 1.341 | 1.341 |
| Z | 2 | 2 |
| Mu (mm-1) | 0.087 | 0.087 |
| F000 | 384.0 | 384.0 |
| F000' | 384.18 |  |
| h,k,lmax | 7,24,14 | 7,24,14 |
| Nref | 5444[ 2806] | 5377 |
| Tmin,Tmax | 0.981,0.991 | 0.905,0.963 |
| Tmin' | 0.973 |  |

| Correction method= # Reported T Limits: Tmin=0.905 Tmax=0.963 AbsCorr = MULTI-SCAN |  |
| --- | --- |

| Data completeness= 1.92/0.99 | Theta(max)= 30.287 |
| --- | --- |

| R(reflections)= 0.0479( 3504) | wR2(reflections)= 0.1348( 5377) |
| --- | --- |

| S = 1.019 | Npar= 253 |
| --- | --- |

The following ALERTS were generated. Each ALERT has the format

**test-name_ALERT_alert-type_alert-level**.

Click on the hyperlinks for more details of the test.


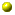
**Alert level C**

[STRVA01_ALERT_4_C](javascript:makeHelpWindow(%22STRVA_01.html%22)) Flack parameter is too small

From the CIF: _refine_ls_abs_structure_Flack -0.500

From the CIF: _refine_ls_abs_structure_Flack_su 0.400

[PLAT340_ALERT_3_C](javascript:makeHelpWindow(%22PLAT340.html%22)) Low Bond Precision on C-C Bonds ............... 0.00411 Ang.

[PLAT360_ALERT_2_C](javascript:makeHelpWindow(%22PLAT360.html%22)) Short C(sp3)-C(sp3) Bond C21 - C22 . 1.43 Ang.


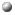
**Alert level G**

[PLAT013_ALERT_1_G](javascript:makeHelpWindow(%22PLAT013.html%22)) N.O.K. _shelx_hkl_checksum Found in CIF ...... Please Check

[PLAT032_ALERT_4_G](javascript:makeHelpWindow(%22PLAT032.html%22)) Std. Uncertainty on Flack Parameter Value High . 0.400 Report

[PLAT883_ALERT_1_G](javascript:makeHelpWindow(%22PLAT883.html%22)) No Info/Value for _atom_sites_solution_primary . Please Do !

[PLAT912_ALERT_4_G](javascript:makeHelpWindow(%22PLAT912.html%22)) Missing # of FCF Reflections Above STh/L= 0.600 20 Note

[PLAT978_ALERT_2_G](javascript:makeHelpWindow(%22PLAT978.html%22)) Number C-C Bonds with Positive Residual Density. 7 Info

0 **ALERT level A** = Most likely a serious problem - resolve or explain

0 **ALERT level B** = A potentially serious problem, consider carefully

3 **ALERT level C** = Check. Ensure it is not caused by an omission or oversight

5 **ALERT level G** = General information/check it is not something unexpected

2 ALERT type 1 CIF construction/syntax error, inconsistent or missing data

2 ALERT type 2 Indicator that the structure model may be wrong or deficient

1 ALERT type 3 Indicator that the structure quality may be low

3 ALERT type 4 Improvement, methodology, query or suggestion

0 ALERT type 5 Informative message, check

| It is advisable to attempt to resolve as many as possible of the alerts in all categories. Often the minor alerts point to easily fixed oversights, errors and omissions in your CIF or refinement strategy, so attention to these fine details can be worthwhile. In order to resolve some of the more serious problems it may be necessary to carry out additional measurements or structure refinements. However, the purpose of your study may justify the reported deviations and the more serious of these should normally be commented upon in the discussion or experimental section of a paper or in the "special_details" fields of the CIF. checkCIF was carefully designed to identify outliers and unusual parameters, but every test has its limitations and alerts that are not important in a particular case may appear. Conversely, the absence of alerts does not guarantee there are no aspects of the results needing attention. It is up to the individual to critically assess their own results and, if necessary, seek expert advice.  **Publication of your CIF in IUCr journals**  A basic structural check has been run on your CIF. These basic checks will be run on all CIFs submitted for publication in IUCr journals (*Acta Crystallographica*, *Journal of Applied Crystallography*, *Journal of Synchrotron Radiation*); however, if you intend to submit to *Acta Crystallographica Section C* or *E* or *IUCrData*, you should make sure that [full publication checks](http://journals.iucr.org/services/cif/checking/checkform.html) are run on the final version of your CIF prior to submission.  **Publication of your CIF in other journals**  Please refer to the *Notes for Authors* of the relevant journal for any special instructions relating to CIF submission. |
| --- |

**PLATON version of 05/12/2020; check.def file version of 05/12/2020**

| **Datablock mo_NAZ01_0m** - ellipsoid plot |
| --- |
| 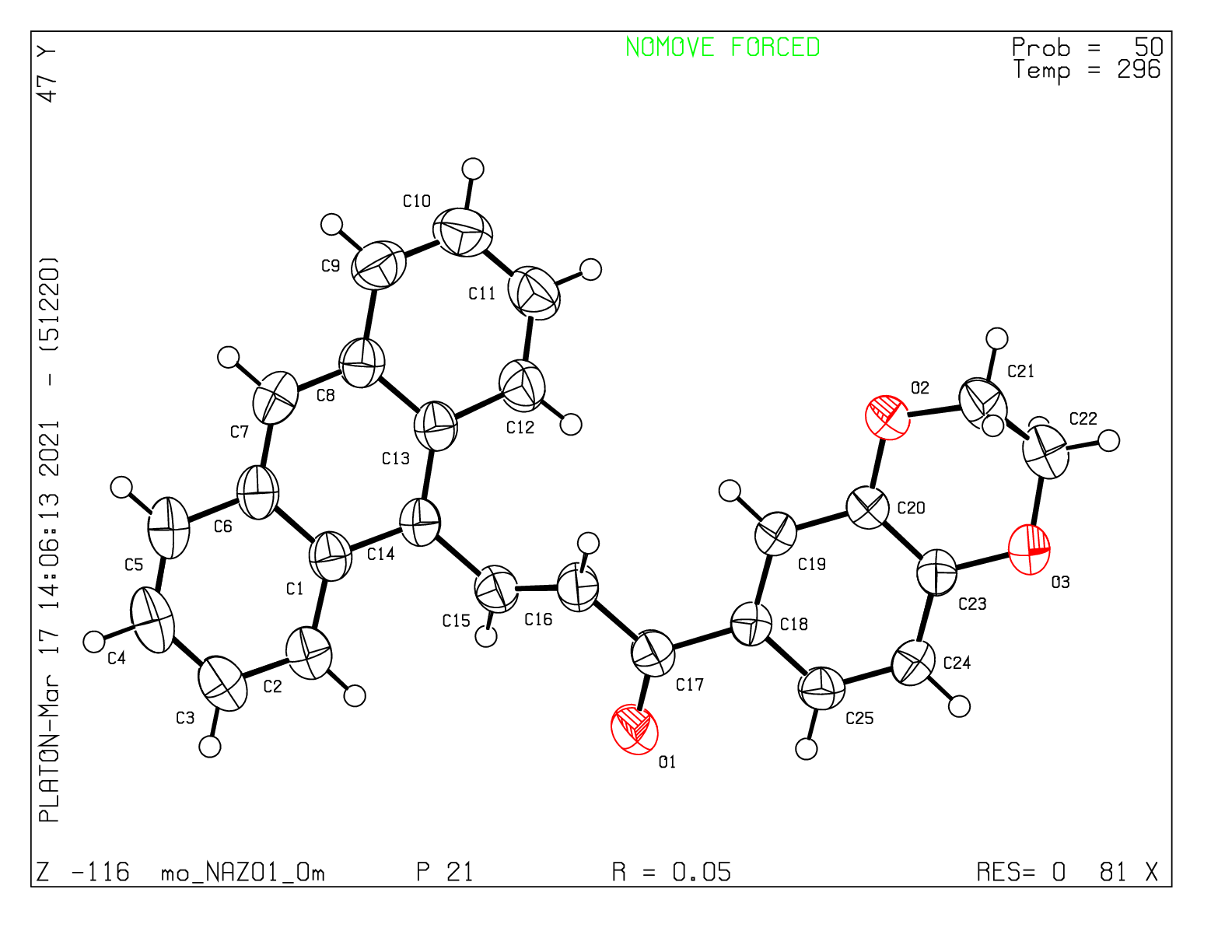 |

**S2 CheckCIF Validation Reports of Anth-2**

**checkCIF/PLATON (basic structural check)**

Structure factors have been supplied for datablock(s) mo_SH04_0m

THIS REPORT IS FOR GUIDANCE ONLY. IF USED AS PART OF A REVIEW PROCEDURE FOR PUBLICATION, IT SHOULD NOT REPLACE THE EXPERTISE OF AN EXPERIENCED CRYSTALLOGRAPHIC REFEREE.

No syntax errors found. [CIF dictionary](http://www.iucr.org/iucr-top/cif/cif_core/definitions/index.html)
Please wait while processing .... [Interpreting this report](http://journals.iucr.org/services/cif/checking/checkcifreport.html)

[Structure factor report](http://checkcif.iucr.org/1BwMGOrlLFLwX/031721141839531352000/ckf.html)

**Datablock: mo_SH04_0m**

| Bond precision: | C-C = 0.0027 A | Wavelength=0.71073 |
| --- | --- | --- |

| Cell: | a=14.5564(11) | b=16.1318(12) | c=16.5002(12) |
| --- | --- | --- | --- |
|  | alpha=90 | beta=90 | gamma=90 |
| Temperature: | 296 K |  |  |

|  | Calculated | Reported |
| --- | --- | --- |
| Volume | 3874.6(5) | 3874.6(5) |
| Space group | P b c a | P b c a |
| Hall group | -P 2ac 2ab | -P 2ac 2ab |
| Moiety formula | C25 H16 O S2 | ? |
| Sum formula | C25 H16 O S2 | C25 H16 O S2 |
| Mr | 396.50 | 396.50 |
| Dx,g cm-3 | 1.359 | 1.359 |
| Z | 8 | 8 |
| Mu (mm-1) | 0.288 | 0.288 |
| F000 | 1648.0 | 1648.0 |
| F000' | 1650.57 |  |
| h,k,lmax | 21,24,24 | 21,24,24 |
| Nref | 6810 | 6803 |
| Tmin,Tmax | 0.848,0.932 | 0.734,0.802 |
| Tmin' | 0.837 |  |

| Correction method= # Reported T Limits: Tmin=0.734 Tmax=0.802 AbsCorr = MULTI-SCAN |  |
| --- | --- |

| Data completeness= 0.999 | Theta(max)= 32.165 |
| --- | --- |

| R(reflections)= 0.0496( 4814) | wR2(reflections)= 0.1789( 6803) |
| --- | --- |

| S = 1.005 | Npar= 253 |
| --- | --- |

The following ALERTS were generated. Each ALERT has the format

**test-name_ALERT_alert-type_alert-level**.

Click on the hyperlinks for more details of the test.


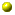
**Alert level C**

[PLAT905_ALERT_3_C](javascript:makeHelpWindow(%22PLAT905.html%22)) Negative K value in the Analysis of Variance ... -2.267 Report


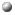
**Alert level G**

[PLAT013_ALERT_1_G](javascript:makeHelpWindow(%22PLAT013.html%22)) N.O.K. _shelx_hkl_checksum Found in CIF ...... Please Check

[PLAT063_ALERT_4_G](javascript:makeHelpWindow(%22PLAT063.html%22)) Crystal Size Possibly too Large for Beam Size .. 0.62 mm

[PLAT883_ALERT_1_G](javascript:makeHelpWindow(%22PLAT883.html%22)) No Info/Value for _atom_sites_solution_primary . Please Do !

[PLAT912_ALERT_4_G](javascript:makeHelpWindow(%22PLAT912.html%22)) Missing # of FCF Reflections Above STh/L= 0.600 8 Note

[PLAT965_ALERT_2_G](javascript:makeHelpWindow(%22PLAT965.html%22)) The SHELXL WEIGHT Optimisation has not Converged Please Check

[PLAT978_ALERT_2_G](javascript:makeHelpWindow(%22PLAT978.html%22)) Number C-C Bonds with Positive Residual Density. 14 Info

0 **ALERT level A** = Most likely a serious problem - resolve or explain

0 **ALERT level B** = A potentially serious problem, consider carefully

1 **ALERT level C** = Check. Ensure it is not caused by an omission or oversight

6 **ALERT level G** = General information/check it is not something unexpected

2 ALERT type 1 CIF construction/syntax error, inconsistent or missing data

2 ALERT type 2 Indicator that the structure model may be wrong or deficient

1 ALERT type 3 Indicator that the structure quality may be low

2 ALERT type 4 Improvement, methodology, query or suggestion

0 ALERT type 5 Informative message, check

| It is advisable to attempt to resolve as many as possible of the alerts in all categories. Often the minor alerts point to easily fixed oversights, errors and omissions in your CIF or refinement strategy, so attention to these fine details can be worthwhile. In order to resolve some of the more serious problems it may be necessary to carry out additional measurements or structure refinements. However, the purpose of your study may justify the reported deviations and the more serious of these should normally be commented upon in the discussion or experimental section of a paper or in the "special_details" fields of the CIF. checkCIF was carefully designed to identify outliers and unusual parameters, but every test has its limitations and alerts that are not important in a particular case may appear. Conversely, the absence of alerts does not guarantee there are no aspects of the results needing attention. It is up to the individual to critically assess their own results and, if necessary, seek expert advice.  **Publication of your CIF in IUCr journals**  A basic structural check has been run on your CIF. These basic checks will be run on all CIFs submitted for publication in IUCr journals (*Acta Crystallographica*, *Journal of Applied Crystallography*, *Journal of Synchrotron Radiation*); however, if you intend to submit to *Acta Crystallographica Section C* or *E* or *IUCrData*, you should make sure that [full publication checks](http://journals.iucr.org/services/cif/checking/checkform.html) are run on the final version of your CIF prior to submission.  **Publication of your CIF in other journals**  Please refer to the *Notes for Authors* of the relevant journal for any special instructions relating to CIF submission. |
| --- |

**PLATON version of 05/12/2020; check.def file version of 05/12/2020**

| **Datablock mo_SH04_0m** - ellipsoid plot |
| --- |
| 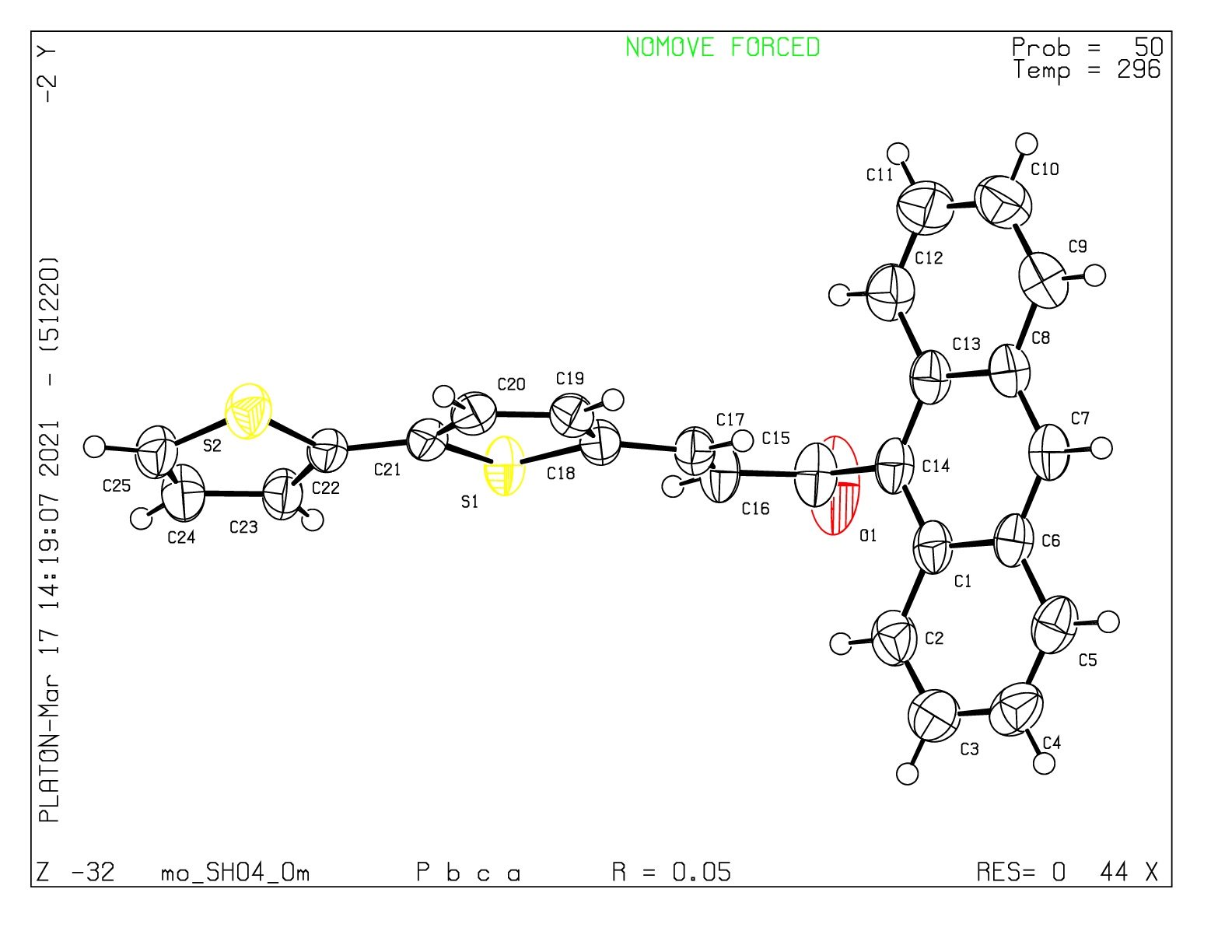 |
