# Supplementary figures and images for: Fused ring effect on optical nonlinearity and structure property relationship of anthracenyl chalcone based push-pull chromophores
Source: PLoS One. 2021 Sep 28;16(9):e0257808. doi: 10.1371/journal.pone.0257808 (PMC8478194; doi:10.1371/journal.pone.0257808)

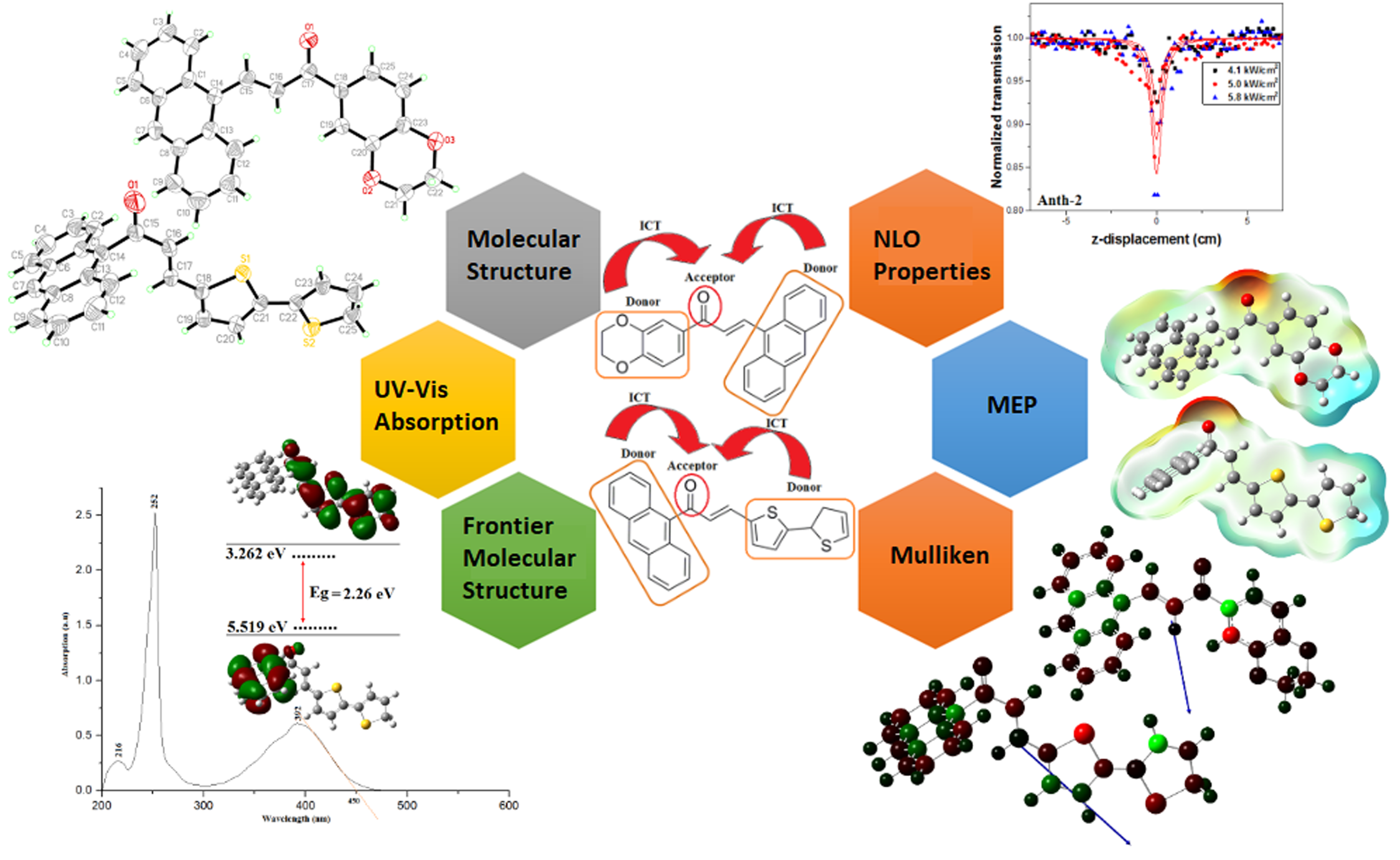

Supplement: S1 Graphical abstract — (TIF) [file pone.0257808.s002.tif]
